# A tale of two symmetrical tails: Structural and functional characteristics of palindromes in proteins

**DOI:** 10.1186/1471-2105-9-274

**Published:** 2008-06-11

**Authors:** Armita Sheari, Mehdi Kargar, Ali Katanforoush, Shahriar Arab, Mehdi Sadeghi, Hamid Pezeshk, Changiz Eslahchi, Sayed-Amir Marashi

**Affiliations:** 1 Bioinformatics Group, School of Computer Science, Institute for Studies in Theoretical Physics and Mathematics (IPM), Tehran, Iran; 2 Computer Engineering Department, Sharif University of Technology, Tehran, Iran; 3 Department of Bioinformatics, Institute of Biochemistry and Biophysics, University of Tehran, Tehran, Iran; 4 National Institute of Genetic Engineering and Biotechnology, Tehran-Karaj Highway, Tehran, Iran; 5 Center of Excellence in Biomathematics, School of Mathematics, Statistics and Computer Science, College of Science, University of Tehran, Tehran, Iran; 6 Faculty of Mathematics, Shahid-Beheshti University, Tehran, Iran; 7 IMPRS-CBSC, Max Planck Institute for Molecular Genetics, Ihnestr. 63-73, Berlin, Germany; 8 DFG-Research Center Matheon, FB Mathematik und Informatik, Freie Universität Berlin, Arnimallee 6, D-14195 Berlin, Germany

## Abstract

**Background:**

It has been previously shown that palindromic sequences are frequently observed in proteins. However, our knowledge about their evolutionary origin and their possible importance is incomplete.

**Results:**

In this work, we tried to revisit this relatively neglected phenomenon. Several questions are addressed in this work. (1) It is known that there is a large chance of finding a palindrome in low complexity sequences (i.e. sequences with extreme amino acid usage bias). What is the role of sequence complexity in the evolution of palindromic sequences in proteins? (2) Do palindromes coincide with conserved protein sequences? If yes, what are the functions of these conserved segments? (3) In case of conserved palindromes, is it always the case that the whole conserved pattern is also symmetrical? (4) Do palindromic protein sequences form regular secondary structures? (5) Does sequence similarity of the two "sides" of a palindrome imply structural similarity? For the first question, we showed that the complexity of palindromic peptides is significantly lower than randomly generated palindromes. Therefore, one can say that palindromes occur frequently in low complexity protein segments, without necessarily having a defined function or forming a special structure. Nevertheless, this does not rule out the possibility of finding palindromes which play some roles in protein structure and function. In fact, we found several palindromes that overlap with conserved protein Blocks of different functions. However, in many cases we failed to find any symmetry in the conserved regions of corresponding Blocks. Furthermore, to answer the last two questions, the structural characteristics of palindromes were studied. It is shown that palindromes may have a great propensity to form α-helical structures. Finally, we demonstrated that the two sides of a palindrome generally do not show significant structural similarities.

**Conclusion:**

We suggest that the puzzling abundance of palindromic sequences in proteins is mainly due to their frequent concurrence with low-complexity protein regions, rather than a global role in the protein function. In addition, palindromic sequences show a relatively high tendency to form helices, which might play an important role in the evolution of proteins that contain palindromes. Moreover, reverse similarity in peptides does not necessarily imply significant structural similarity. This observation rules out the importance of palindromes for forming symmetrical structures. Although palindromes frequently overlap with conserved Blocks, we suggest that palindromes overlap with Blocks only by coincidence, rather than being involved with a certain structural fold or protein domain.

## Background

Symmetry of shapes is something that is found everywhere in nature. Human beings have always been attracted to symmetrical properties of natural phenomena, and their interest is reflected in art.

Creation of symmetrical sentences (i.e. "palindromes") goes back to at least 20 centuries ago. The Sator Square [1] contains probably the oldest known palindrome, which shows the Latin sentence "SATOR AREPO TENET OPERA ROTAS". Note that the word "tenet" is a palindrome itself. Since then, many other famous palindromic sentences have been constructed in different languages.

With the progress of molecular biology in the 20th century, a new level of symmetry was discovered in nature. From the study of restriction endonucleases in the 60's and the early 70's [2-5], it became clear that a certain type of palindrome, i.e. reverse palindrome, occur in DNA sequences. Restriction enzymes recognition sites which exist in double-stranded and not single stranded DNA, are usually "palindromic". For example:

G A A T T C

C T T A A G

is an example of a palindromic sequence recognized by restriction enzyme *Eco*RI. They are usually referred to as "reverse palindromes", because the sequence and polarity of both strands of the DNA molecule define their palindromic nature. Several studies suggest that reverse palindromes (or for simplicity, "palindromes") are statistically under-represented in some genomes [6-10], presumably because of the existence of restriction endonucleases in the host cells. Palindromic sequences are known to have roles in DNA replication [11] and RNA transcription [12].

In proteins, palindromes appear in a polypeptide chain. For example, the hypothetical protein sequence:

PQRSRQP

is a palindromic sequence. **PQR **and **RQP **will be referred to as the "sides" of this palindrome, while **S **will be called the "linker" (see Methods).

Since the original suggestion of the existence of important palindromic sequences in proteins [13-15], or simply "palindromic peptides", relatively little effort has been made to find the significance of such sequences. Inverse sequence similarity of proteins is not an exception by any means [16,17], and some studies suggest that palindromes may appear in protein sequences more frequently than is expected by chance [18,19]. Many have tried to find a relationship between palindromic sequences and protein structure. It has been suggested, directly or indirectly, that palindromic sequences are important for the structure and/or function of several classes of proteins, including DNA binding proteins [15,18,20], the Rhodopsin family and ion channels [13], prions [21,22], metal binding proteins [23] and receptors [24]. Some synthetic proteins which have structural characteristics as native proteins, as in the case of collagen protein model [25], can be added to this list.

In this work, we try to address the question of why palindromes are so frequent in proteins and to see if they have specific functions. In addition, we try to find a possible relationship between the symmetry of the sequence and the structure.

## Results and Discussion

### Linguistic complexity of palindromes

Ohno, [15] in one of his short papers on the importance of palindromic sequences in proteins, suggested that H1 histone is rich with palindromes only because of the high frequency of alanine and lysine (48% of all residues) in its sequence. In an effort to pursue this proposal, we try to test whether palindromes are a result of a biased amino acid usage, or in other words, a result of "low complexity". We use linguistic complexity (LC) as a measure of complexity of sequence. LC takes values between 0 and 1. The lower the LC value, the lower the complexity of the sequence. We compare the distribution of LC values in real palindromes and randomly generated palindromes, as explained in Materials and Methods.

Table 1 summarizes the results of this comparison. The Mann-Whitney test was used to assess the significance of differences observed between medians of distributions. The results clearly suggest that the linguistic complexity of real protein palindromes is significantly lower than what is observed in randomly constructed palindromes.

**Table 1:** Comparison of the distribution of linguistic complexity (LC) values in real vs. randomly generated palindromes.

Length(*X*), Length(*Y*)	Average LC of the real set	Average LC of the random set	Level of significance in Mann-Whitney test
3,0	0.769	0.841	*
3,1	0.805	0.869	**
3,2	0.867	0.893	*
4,0	0.761	0.869	**
4,1	0.831	0.886	**
4,2	0.889	0.903	**
4,3	0.897	0.913	**
5,0	0.628	0.888	**
5,1	0.687	0.899	**
5,2	0.866	0.911	*
5,3	0.828	0.918	**
5,4	0.929	0.928	NS

For a definition of *X *and *Y *in a palindrome please see Methods. NS: non-significant difference; *: significant at the level of 0.05; **: significant at the level of 0.01.

### Palindromic peptides and their probable functional roles

Palindromic sequences are known to be present in a variety of proteins [14,18], and different functions have been proposed to be associated with them. We tried to find roles of palindromic sequences in a systematic way, by comparing palindromes with conserved sequences recorded in the Blocks database [26,27].

From our protein dataset of 1094 proteins, only 373 contained at least one reported Block. From these proteins, 54 Blocks overlapped with palindromic sequences in the corresponding proteins. These Blocks are listed in a file submitted with this article (see Additional file 1). It was interesting to find that a variety of functions were possibly associated with some palindromic sequences.

**Figure 1:**
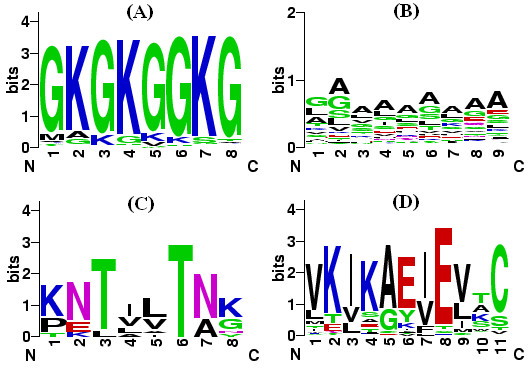
**Examples of Blocks that contain palindromic peptides.** The y-axis shows bits of information [52] in each position of the corresponding palindrome. (A) Block corresponding to the palindrome in PDB ID 1tzy chain D; (B) Block corresponding to the palindrome in PDB ID 1a99 chain A; (C) Block corresponding to the palindrome in PDB ID 1agj chain A; and (D) Block corresponding to the palindrome in PDB ID 1yz7 chain A.

Figure 1 shows examples of Blocks that contain palindromic protein segments. Conserved palindromes in Figures 1A and 1B are presumably the result of low sequence complexity. As mentioned before, such sequences are prone to produce palindromes. The Block in Figure 1C clearly includes a palindromic consensus. This Block might have evolved from a palindromic ancestor with serine protease activity. Finally, there are palindromes in conserved Blocks like that in Figure 1D, which seem to be completely accidental.

### Is the symmetry of palindromic sequences reflected in their structures?

Can palindromic protein sequences help in formation of structurally symmetrical folds? For answering this question one should test whether symmetry of palindromic peptides is reflected in their structure.

While the 3D structure of reverse palindromes in double-stranded DNA is symmetrical, there is much debate about the structural similarity of protein sequences which have reverse similarity. One might expect that reversing the sequence would result in folds that are mirror-images of the original fold [28]. However, there exists theoretical and experimental evidences that sequence reversing results in the same rather than the mirror folding, presumably due to the fact that both native and reverse proteins have the same amino acid compositions and/or similar hydrophobic-hydrophilic patterns [23,29,30]. Evidence suggesting reverse peptide sequences result in different structures has also been presented in the literature. From analysis of Retro-inverso peptides (reversed peptides consisting of _D_ instead of _L_ amino acids), it became clear that, with few exceptions [31,32], these peptides behave very similarly and are even recognized by the same antibodies. Contrary to these, reversed peptides generally behave differently [33-38]. Moreover, many research groups have reported that reversing a sequence can change its fold [39,40], or even extinguish its folding ability [41]. In general, the sequence similarity of reverse sequences may not imply a significant structural similarity [17,42].

We studied the structural characteristics of the two sides of palindromic sequences. We considered very short peptides with perfect reverse-similarity in sequence. This condition assured that differences in structure cannot be due to differences in sequences. Since we did not allow long linker sequences between the two sides of the palindromes, peptide segments coded by the two sides are close to each other in space and therefore, are expected to form their fold in a similar environment.

If both sides of a palindrome appear in the same secondary structure, they will be considered structurally similar. Therefore, we grouped our palindromes into three classes: palindromes whose both sides have α-helical structure: "all-alpha", palindromes whose both sides have β-sheet structure: "all-beta", and other palindromes: "others".

Among the 980 palindromic sequences in our dataset, 120 (12.2%) were "all-alpha", 7 (0.7%) were "all-beta", and the remaining 853 (87.1%) were classified as "others". Among the 489720 randomly sequences occurring in the proteins, 16449 (3.4%) were "all-alpha", 2973 (0.6%) were "all-beta", and the remaining 470248 (96%) fall into "others" class. The results suggest that palindromes have significantly greater tendency to appear in α-helices compared to random sequences. There seems to be only a weak preference for palindromic sequences to appear in strands.

It is generally accepted that sequences of natural proteins are far from being random. It has been shown that, unless amino acid composition is restrained [43] or a binary patterning of polar and non-polar amino acids is defined [44,45], proteins with random sequences rarely form secondary structures [46]. Evidently, decreased amino acid composition complexity or binary patterning of polar and non-polar amino acids increases the chance for palindrome formation. Furthermore, it has been shown that proteins formed from repeats of non-natural peptides and palindromic peptides can form secondary structures in solution [47]. Our results suggest that, even compared to (randomly-selected) natural protein fragments, palindromes have a greater tendency to form at least α-helices, which might be related to their frequent appearance in proteins. This finding might be of great importance for designing *de novo *secondary structures. Certainly, all palindromes do not form helices. It might be interesting to investigate other factors that alter the tendency of palindromic peptides to form regular secondary structures like helices.

We also compared the structural similarity (RMSD) between the two palindrome sides to the corresponding value in a set of randomly selected protein fragments. Since a few atoms in the PDB structures of randomly selected fragments were often missing, we additionally compared the Cα trace of those fragments. For the palindromic sequences, we compared the structures of backbones of the two sides of a palindrome, and also the structure of the backbone of the left side with the structure of the backbone of mirror image of the right side. In each case, an RMSD value was calculated to assess structural similarity. In order to see whether this alignment is significantly "good", we compare the RMSD values to the same values computed for randomly selected protein fragments of the same length. A good structural similarity of the two sides must result in smaller RMSD values for the palindrome sides as compared to randomly selected fragments.

Table 2 summarizes the results of the comparisons. Only a small number of palindromes showed significantly smaller RMSD values and the average RMSD of palindromes is almost in the middle of the RMSD values for random fragments. This implies that the two sides of a palindrome do not in general show an "exceptional" structural similarity. In other words, reverse similarity at the sequence level in proteins is not necessarily reflected at the level of structure.

**Table 2:** Summary of the RMSD comparison for the two sides of each palindrome. See text for more details.

	Total number of analyzed palindromes	No. of palindromes with *P *< 0.05	No. of palindromes with *P *< 0.01	Average RMSD of palindrome sides (Å)	Ratio of random fragments with smaller RMSD values
Structural alignment of the two sides (backbone atoms)	352	9	0	1.995	0.532
Structural alignment of one side with the mirror image of the two sides (backbone atoms)	352	14	4	2.123	0.527
Structural alignment of the two sides (Cα only)	561	10	0	0.964	0.487
Structural alignment of one side with the mirror image of the two sides (Cα only)	561	13	2	0.967	0.487

We also focused on the short conserved palindrome shown in Figure 1C. The values of *P *for none of the four comparisons for this palindrome were less than 0.66. Furthermore, the two sides of this conserved palindrome have different secondary structures. This example confirms that the reverse similarity of the protein sequence is probably not reflected in the structure. Altogether, one may conclude that palindromic peptides can hardly help in forming symmetrical structures in proteins and this certainly is not a reason for their prevalence in proteins.

## Conclusion

We suggest that the puzzling abundance of palindromic sequences in proteins is mainly due to their frequent concurrence with low-complexity protein regions, rather than a global role in the protein function. In addition, palindromic sequences show a relatively high tendency to form helices, which might play an important role in the evolution of proteins that contain palindromes. Moreover, reverse similarity in peptides does not necessarily imply significant structural similarity. This observation rules out the importance of palindromes for forming symmetrical structures.

It is not unusual to find palindromes within functionally conserved protein Blocks. However, the conserved Blocks have very different functions, which suggest that palindromes overlap with Blocks only by coincidence, rather than being involved with a certain structural fold or domain. In addition, many of the conserved patterns are not "symmetrical", which confirms that Blocks and protein palindromes overlap accidentally.

## Methods

### Dataset

We obtained sequences for all the proteins in the Protein Data Bank, PDB [48] in FASTA format (30 December 2006). This dataset contained 38224 proteins. Using the PISCES culling server [49,50], the sequence dataset was filtered to obtain sequences with mutual similarity of less than 30%, with structure resolution <2 Å. The final dataset contained 1094 proteins. The structures of these proteins were obtained from PDB.

### Palindrome definition

We define palindrome to be any sequence as *XYX*^*R*^, in which *X*, *Y *and *X*^*R *^are strings of the 20 standard amino acids, and *X*^*R *^is the reverse of string *X*. In this palindrome, *X *and *X*^*R *^will be referred to as the palindrome "sides", while *Y *is the "linker". Length of a sequence *S *will be shown by |*S*|.

In this study we have considered those palindromes for which |*X*| ≥ |*Y*| ≥ 0 and |*X*| = |*X*^*R*^| ≥ 3. This means that there might be no linker sequence between the sides of a palindrome. In addition, we have assumed that in the standard single-letter representation of amino acids, D = E and K = R. This assumption has helped us to obtain more palindromes so as to be able to perform statistical tests.

In our palindrome dataset, many palindromes are substrings of other palindromes. For example, ABCDDCBA is a palindrome with the side length of four and linker length of zero. One may also consider it as a palindrome of side length three and linker length of two. However, in this study only the palindrome with the maximum side length was included in our palindrome dataset. We also removed 6-His tag sequences from our dataset. These sequences are added artificially to the N-terminal of recombinant proteins to facilitate their purification and are not present in native proteins.

### Analysis of the complexity of palindromic sequences

For each palindromic sequence, we calculated its corresponding "linguistic complexity", LC [51], which is defined as the ratio of the number of distinct substrings present in the string of interest to the maximum possible number of substrings for a string of the same length over the same alphabets.

If palindromes principally appear as low-complexity protein segments, the complexity scores of these sequences will be significantly smaller than that of random palindromic sequences. Therefore, for a palindrome family such as *XYX*^*R*^, with certain values for |*X*| and |*Y*|, we constructed 10000 random palindromes. For each random palindrome, two random sequences with lengths |*X*| and |*Y*| were constructed, based on the average frequencies of the twenty amino acids in our dataset. The sequences were joined to create *X*+*Y*+*X*^*R*^. Finally, the LC distribution of real palindromes was compared to the LC distribution of randomly generated palindromes.

### Finding overlaps between palindromes and functional sites

To investigate possible overlaps between conserved Blocks [26,27] and palindromic sequences, we first tried to identify known Blocks of proteins in our dataset. If Blocks were found, we then determined whether the palindromes in the protein fall within the Blocks (or alternatively, the Blocks fall within any of the palindromes).

In order to visualize Blocks and get an overview of their conservation, WebLogo [52] was used to construct graphical representations of the patterns.

### Analysis of three dimensional structures of palindromic peptides

It is rational to assume that protein segments with the same secondary structures may show greater structural similarity, regardless of their sequence similarity. Therefore, for the analysis of 3D structure of palindromic sequences, we first determined the secondary structure of palindromic segments using DSSP software [53]. Then, based on secondary structure data, we categorized palindromes into "all-alpha" (where the whole structure was in α-helix), "all-beta" (where the whole structure was in β-sheet), and "others". Finally, we compared the structural coordinates of each palindromic peptide with a set of randomly selected peptides in the same category.

Some reports suggest that reversing the sequence will result in the same protein fold [23,29,30]. To test this, we compared the conformation of backbone atoms in the two palindrome sides. It has also been suggested that reversing the sequence can result in a structure which is the mirror image of the original structure [28]. Therefore, we additionally compared the backbone of one palindrome side with the mirror image of the other side. Both of these comparisons were performed for all backbone atoms and for their Cα traces.

For each comparison an RMSD value was calculated. In order to see whether there was a significantly small value, from each protein in the dataset we chose 10 random fragments of the same length (i.e. with the length of 2|*X*|+|*Y*|) and considered the first |*X*| amino acids as the left side of the "pseudo-palindrome" and the last |*X*| amino acids as the right side, and then the structural comparison was performed. If the RMSD for structural alignment of the two palindrome sides is small (i.e. the two sides were structurally similar), then only a small fraction, say *P*, of randomly selected fragments will show a smaller value.

### Statistical analysis

We used Mann-Whitney test for testing the significance of differences between medians of distributions. This test is useful when the two distributions are skewed and cannot be approximated by a normal distribution [54].

## Abbreviations

LC: linguistic complexity; RMSD: Root mean square deviation; PDB: Protein Data Bank.

## Authors' contributions

S–AM presented the original idea. S–AM, MS, HP, CE and AK participated in the design of the methods and led the project. AS, MK, AK and SA implemented the methods. The manuscript was drafted by S–AM. All authors contributed to the discussions for the improvement of the original draft and approved the final manuscript.

## Supplementary Material

Additional file 1 Conserved Blocks that overlap with palindromes in proteins. Click here for file
