# Supplementary figures and images for: A tale of two symmetrical tails: Structural and functional characteristics of palindromes in proteins
Source: BMC Bioinformatics. 2008 Jun 11;9:274. doi: 10.1186/1471-2105-9-274 (PMC2474621; doi:10.1186/1471-2105-9-274)

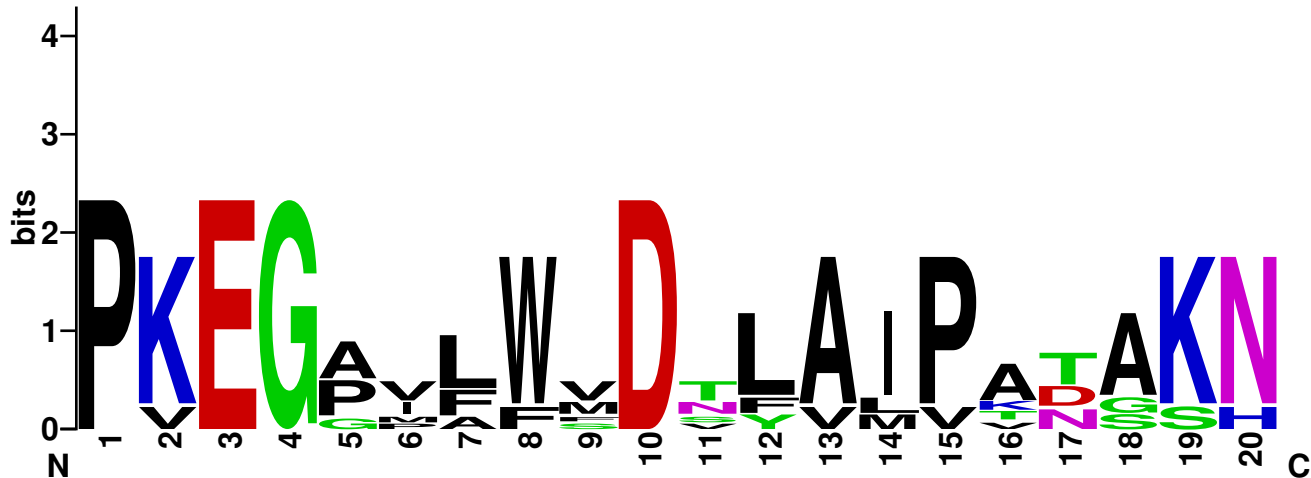

Supplement: Additional file 1 — Conserved Blocks that overlap with palindromes in proteins. [file 1471-2105-9-274-S1.zip › Logo/1A99A.pdf]

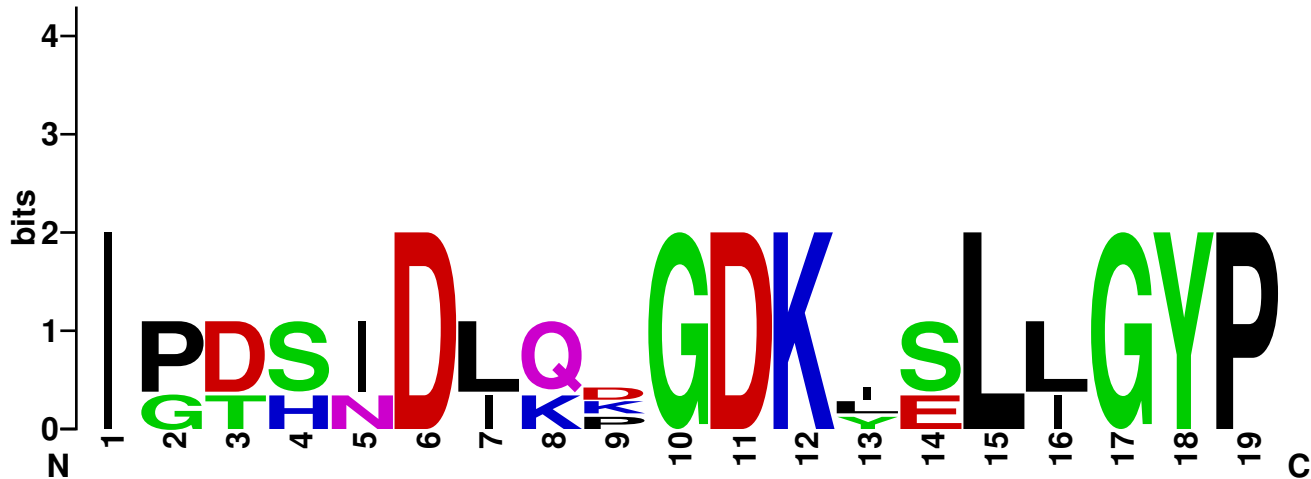

Supplement: Additional file 1 — Conserved Blocks that overlap with palindromes in proteins. [file 1471-2105-9-274-S1.zip › Logo/1AGJA, DLKDGDKLE.pdf]

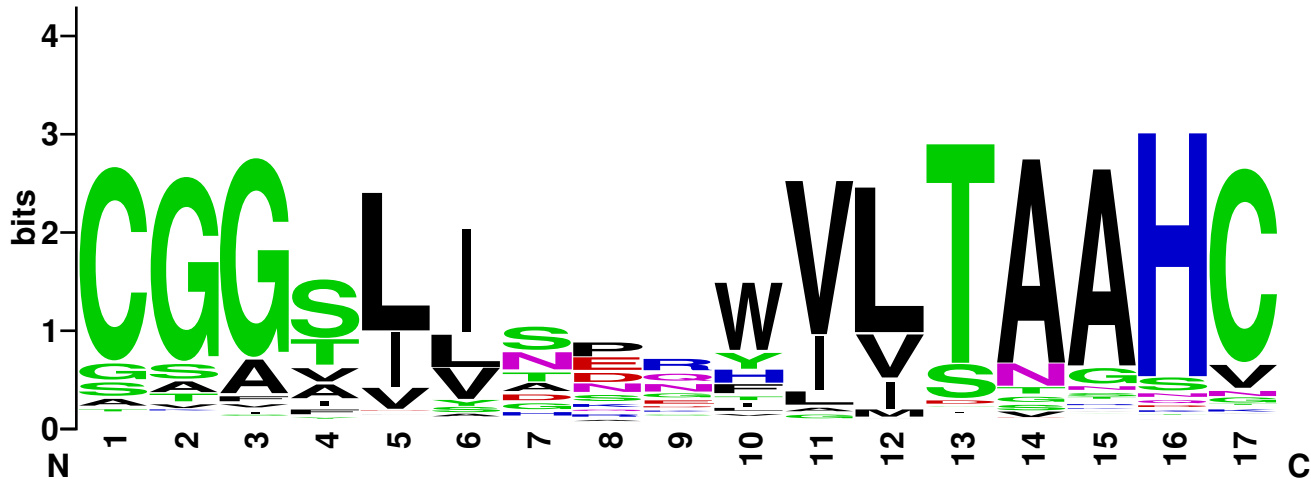

Supplement: Additional file 1 — Conserved Blocks that overlap with palindromes in proteins. [file 1471-2105-9-274-S1.zip › Logo/1AGJA, KNTVLTNR.pdf]

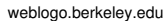

Supplement: Additional file 1 — Conserved Blocks that overlap with palindromes in proteins. [file 1471-2105-9-274-S1.zip › Logo/1B7YB.pdf]

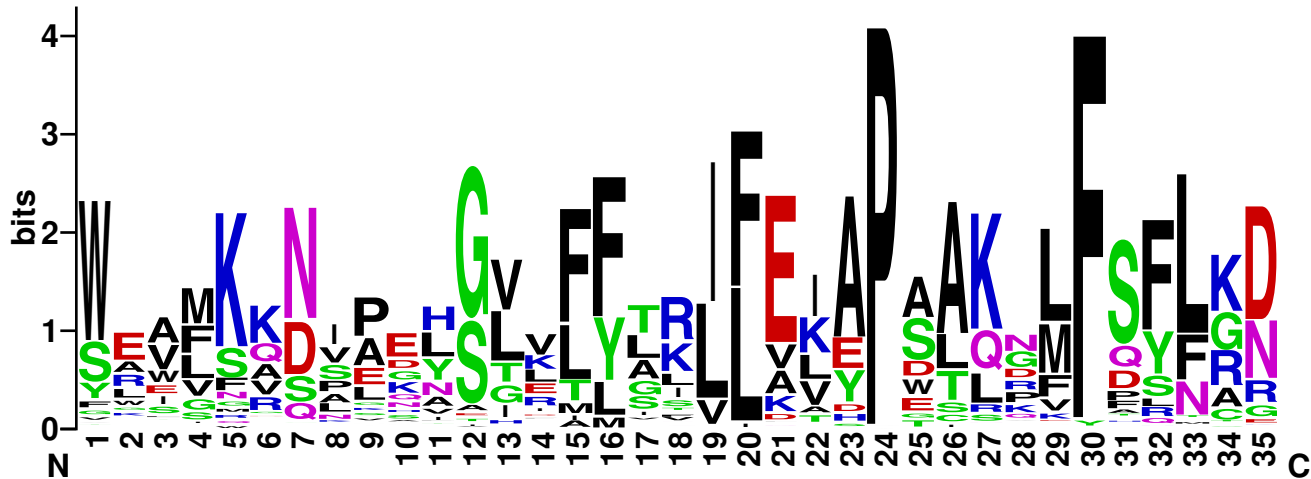

Supplement: Additional file 1 — Conserved Blocks that overlap with palindromes in proteins. [file 1471-2105-9-274-S1.zip › Logo/1BINA.pdf]

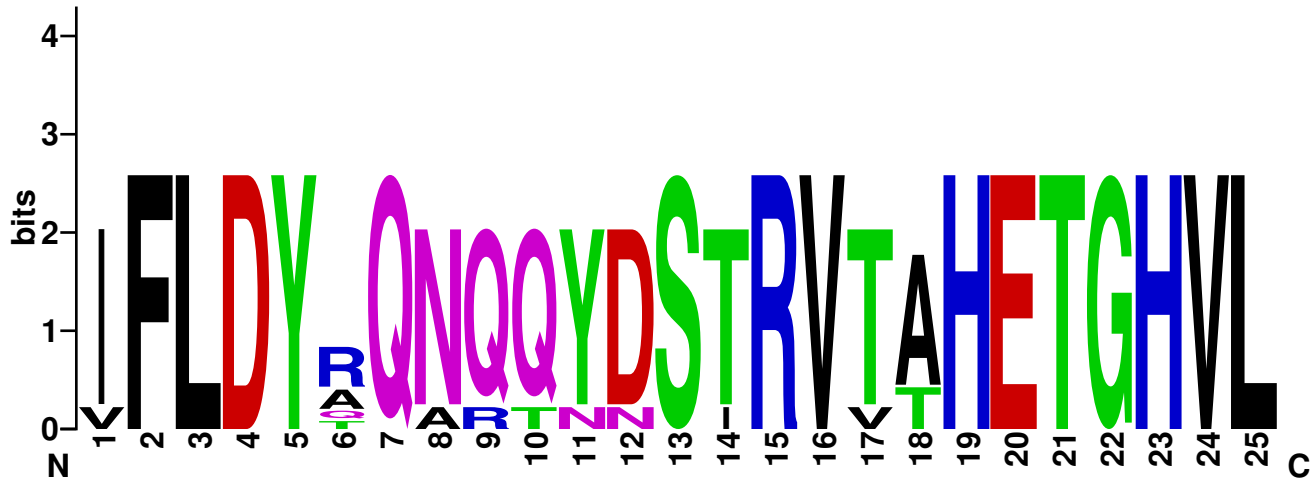

Supplement: Additional file 1 — Conserved Blocks that overlap with palindromes in proteins. [file 1471-2105-9-274-S1.zip › Logo/1C7KA.pdf]

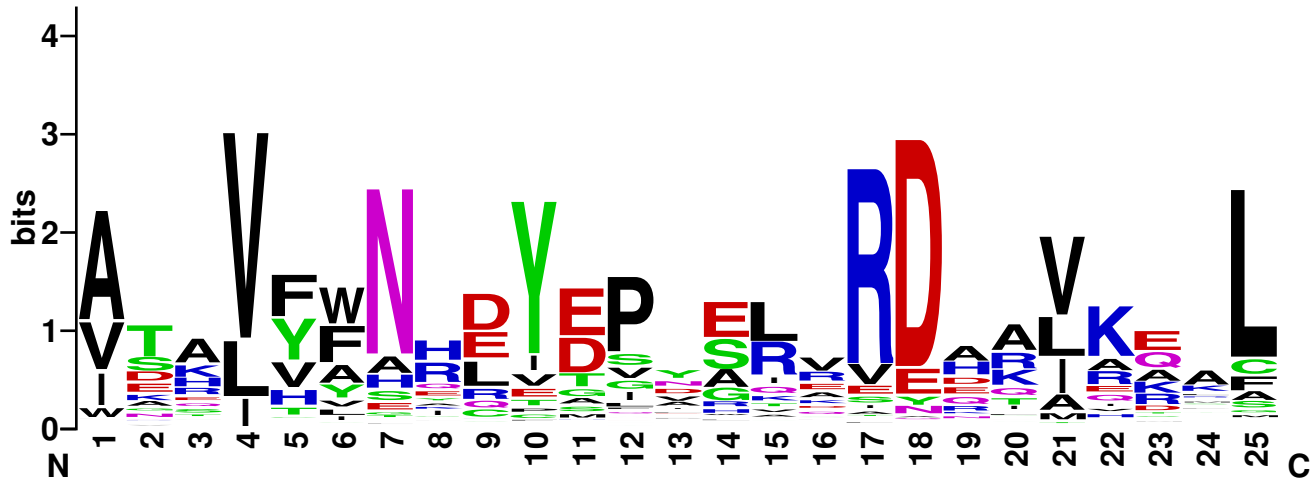

Supplement: Additional file 1 — Conserved Blocks that overlap with palindromes in proteins. [file 1471-2105-9-274-S1.zip › Logo/1DNPA.pdf]

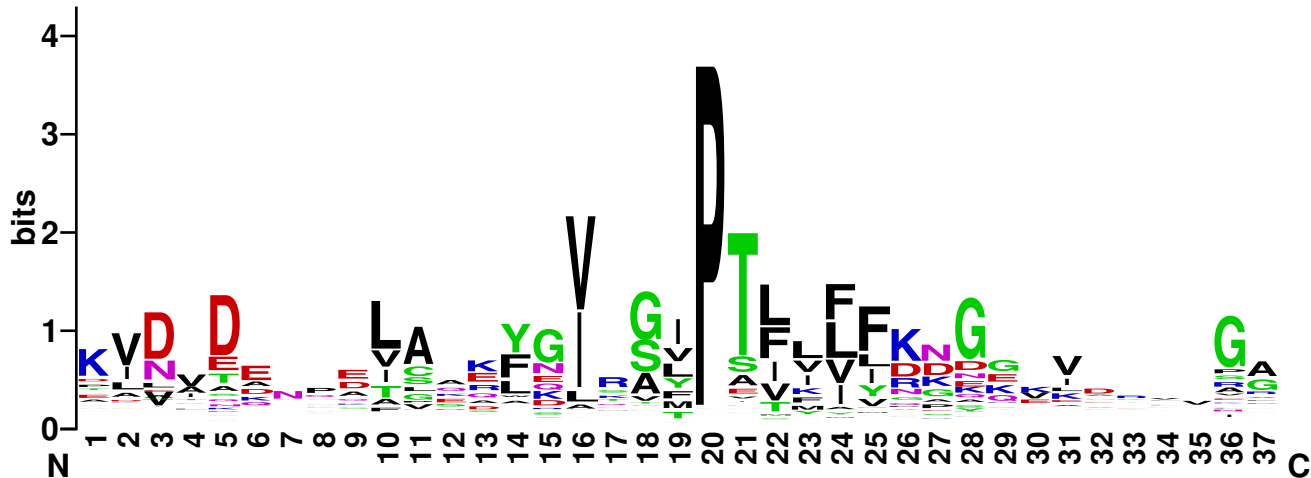

Supplement: Additional file 1 — Conserved Blocks that overlap with palindromes in proteins. [file 1471-2105-9-274-S1.zip › Logo/1EP7A.pdf]

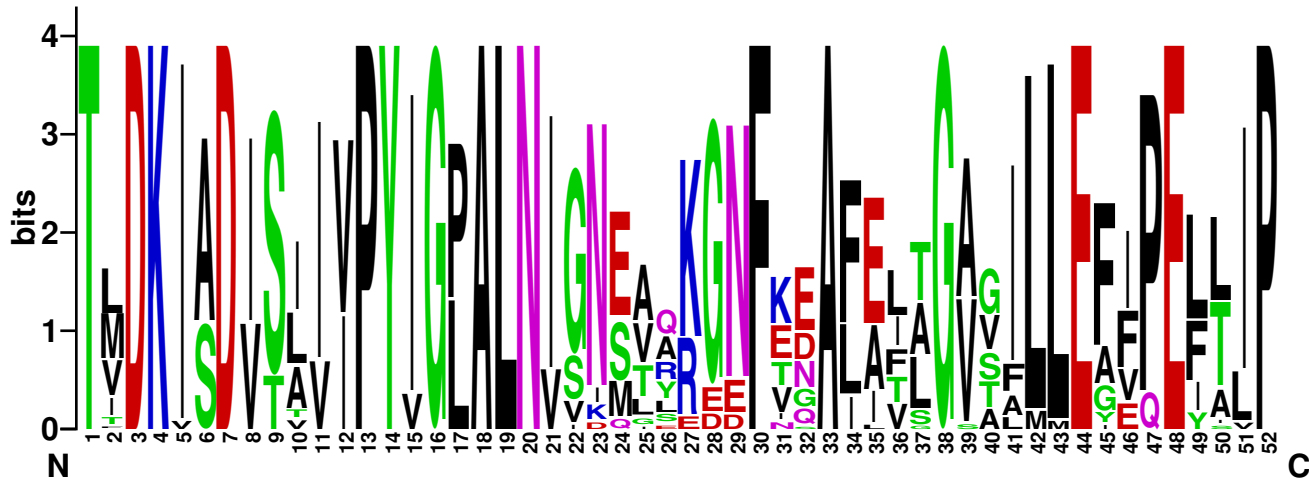

Supplement: Additional file 1 — Conserved Blocks that overlap with palindromes in proteins. [file 1471-2105-9-274-S1.zip › Logo/1EPWA.pdf]

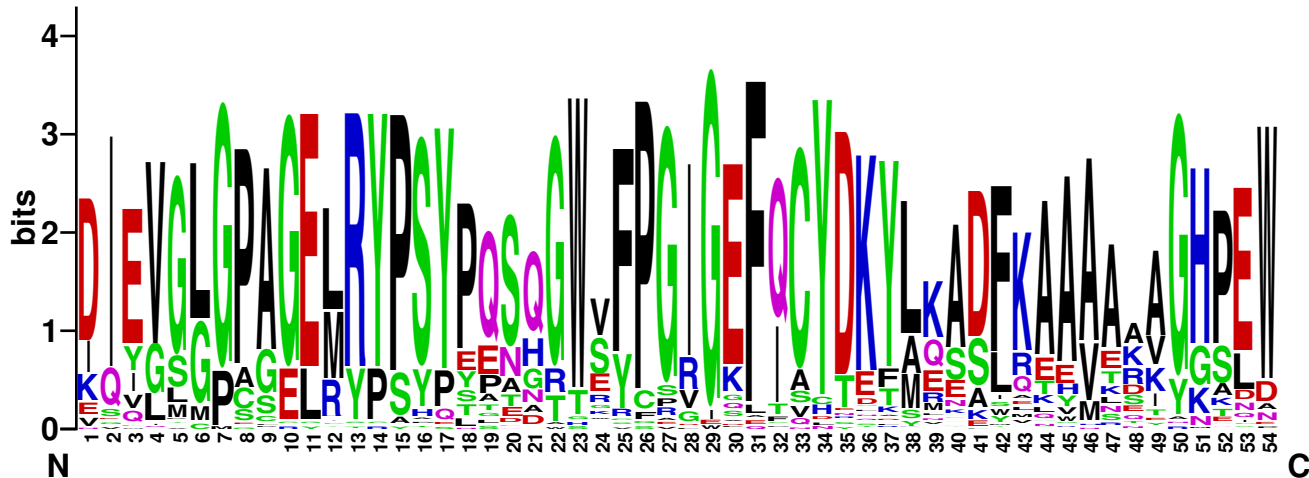

Supplement: Additional file 1 — Conserved Blocks that overlap with palindromes in proteins. [file 1471-2105-9-274-S1.zip › Logo/1FA2A.pdf]

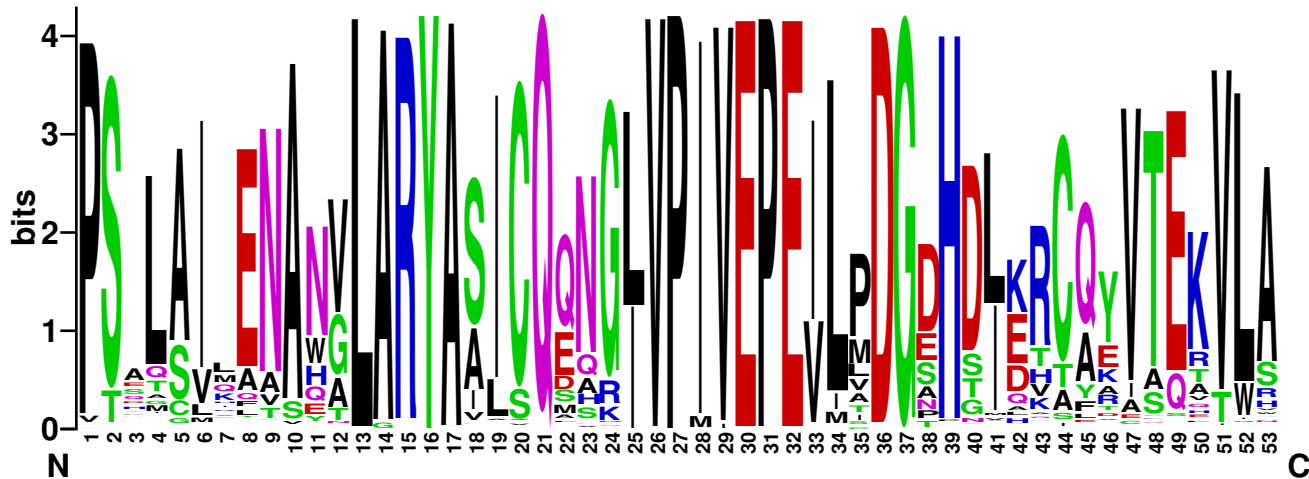

Supplement: Additional file 1 — Conserved Blocks that overlap with palindromes in proteins. [file 1471-2105-9-274-S1.zip › Logo/1FDJA.pdf]

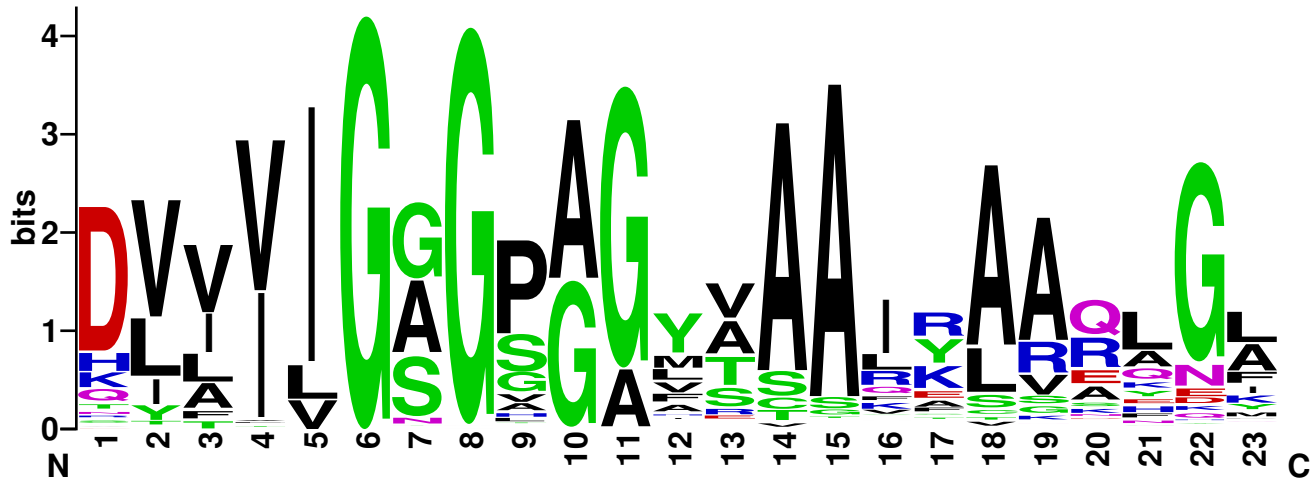

Supplement: Additional file 1 — Conserved Blocks that overlap with palindromes in proteins. [file 1471-2105-9-274-S1.zip › Logo/1GESA.pdf]

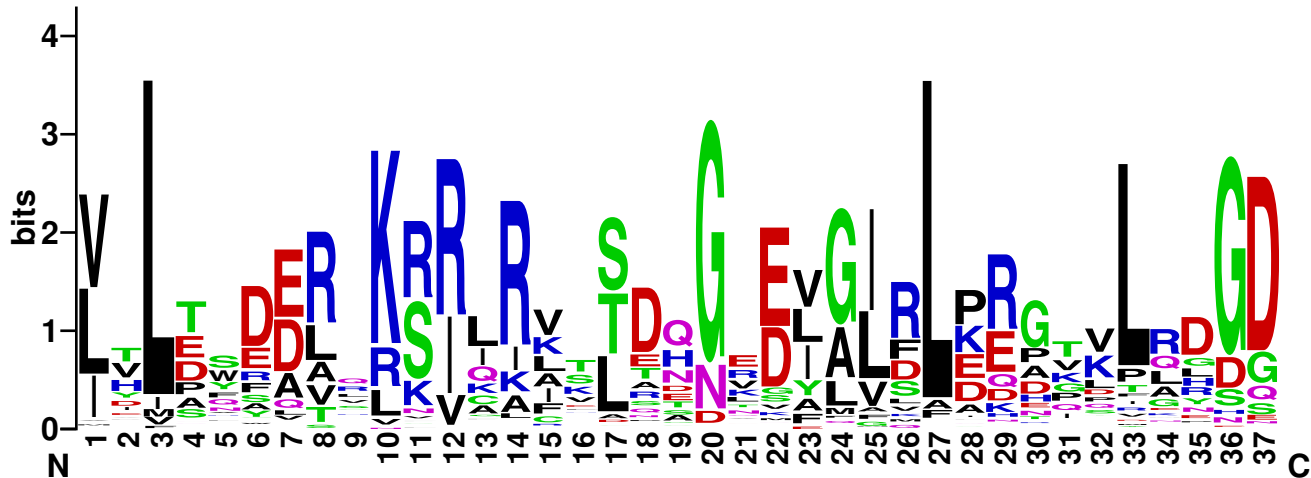

Supplement: Additional file 1 — Conserved Blocks that overlap with palindromes in proteins. [file 1471-2105-9-274-S1.zip › Logo/1GMUA.pdf]

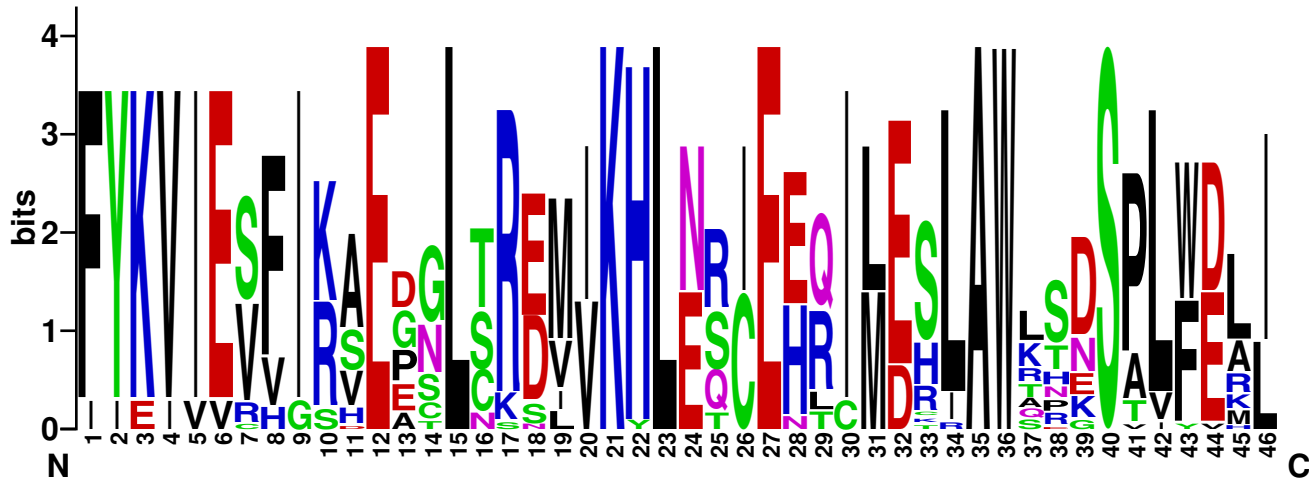

Supplement: Additional file 1 — Conserved Blocks that overlap with palindromes in proteins. [file 1471-2105-9-274-S1.zip › Logo/1GUXA.pdf]

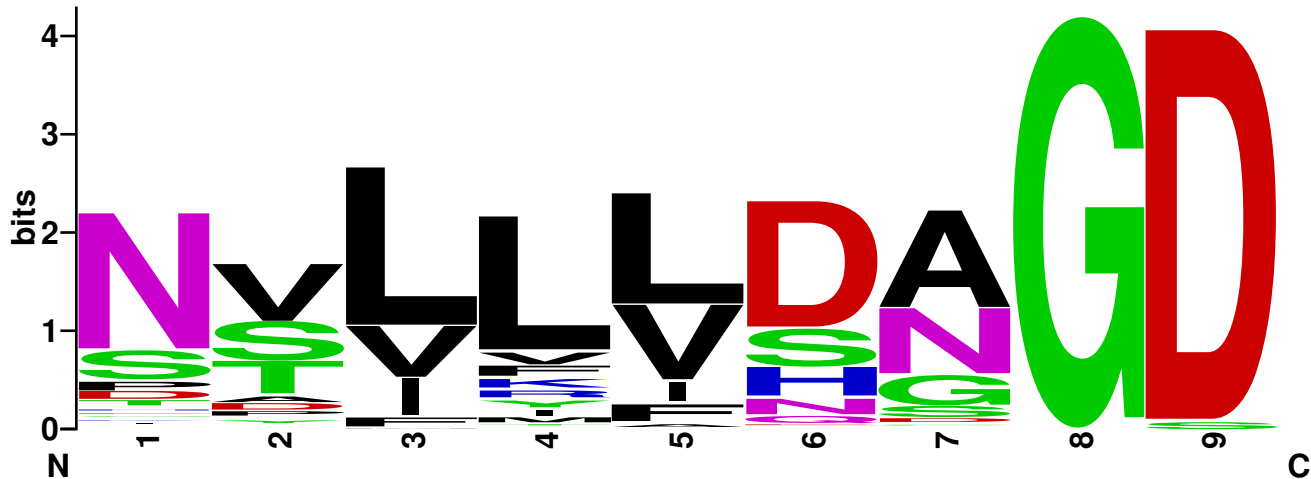

Supplement: Additional file 1 — Conserved Blocks that overlap with palindromes in proteins. [file 1471-2105-9-274-S1.zip › Logo/1HP1A.pdf]

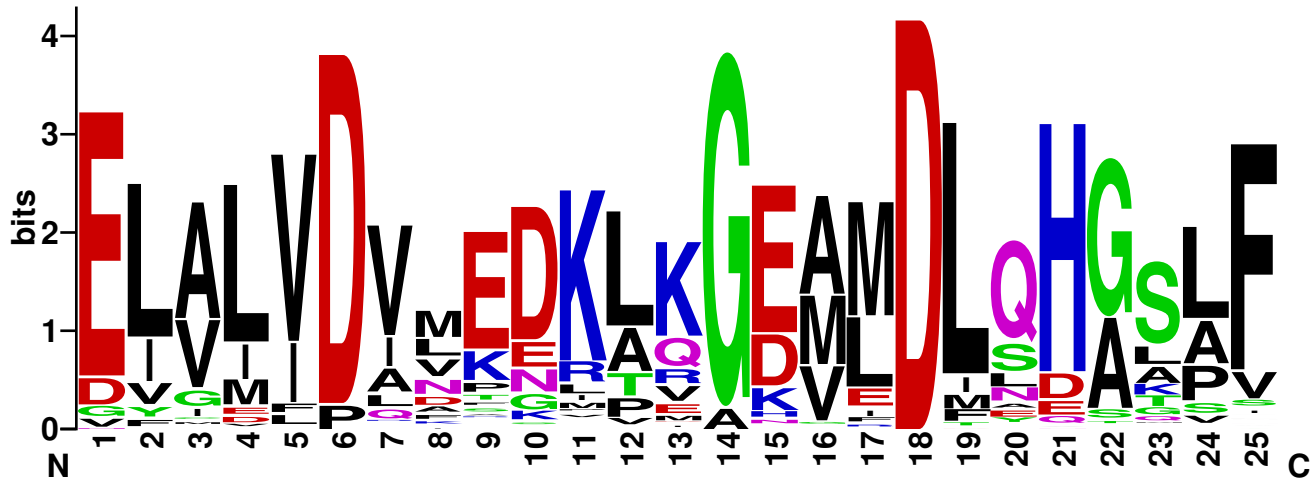

Supplement: Additional file 1 — Conserved Blocks that overlap with palindromes in proteins. [file 1471-2105-9-274-S1.zip › Logo/1HYHA, ADQIDFQDA.pdf]

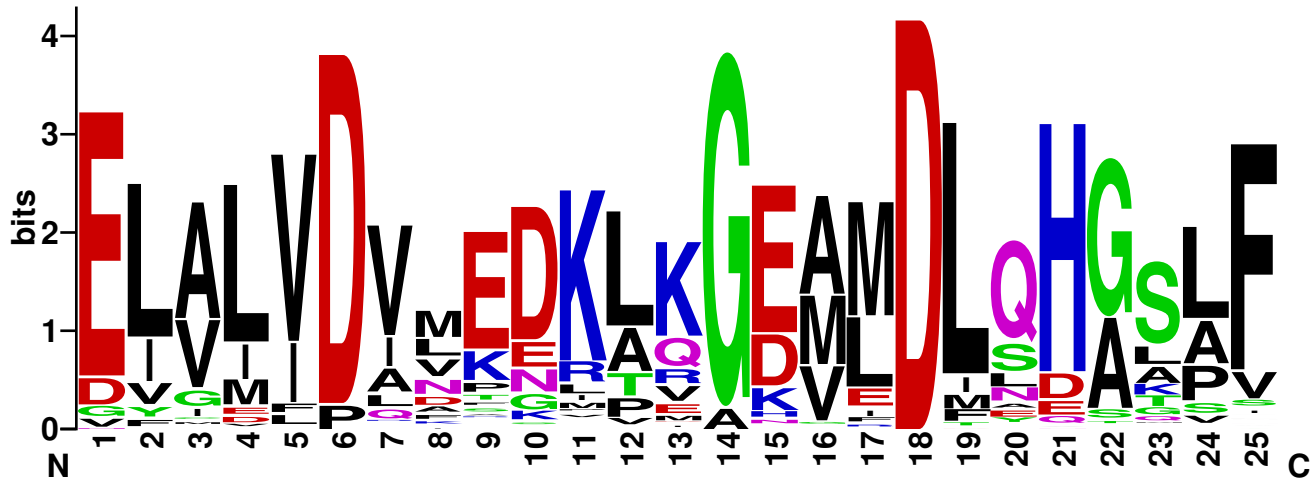

Supplement: Additional file 1 — Conserved Blocks that overlap with palindromes in proteins. [file 1471-2105-9-274-S1.zip › Logo/1HYHA, EAKVKAD.pdf]

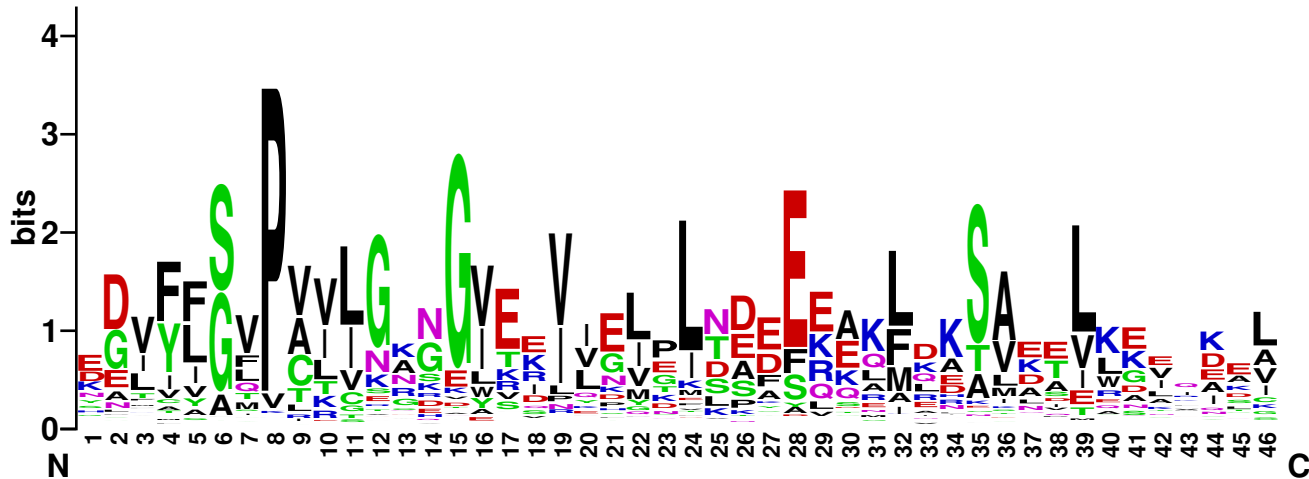

Supplement: Additional file 1 — Conserved Blocks that overlap with palindromes in proteins. [file 1471-2105-9-274-S1.zip › Logo/1HYHA, ETTLDLTTD.pdf]

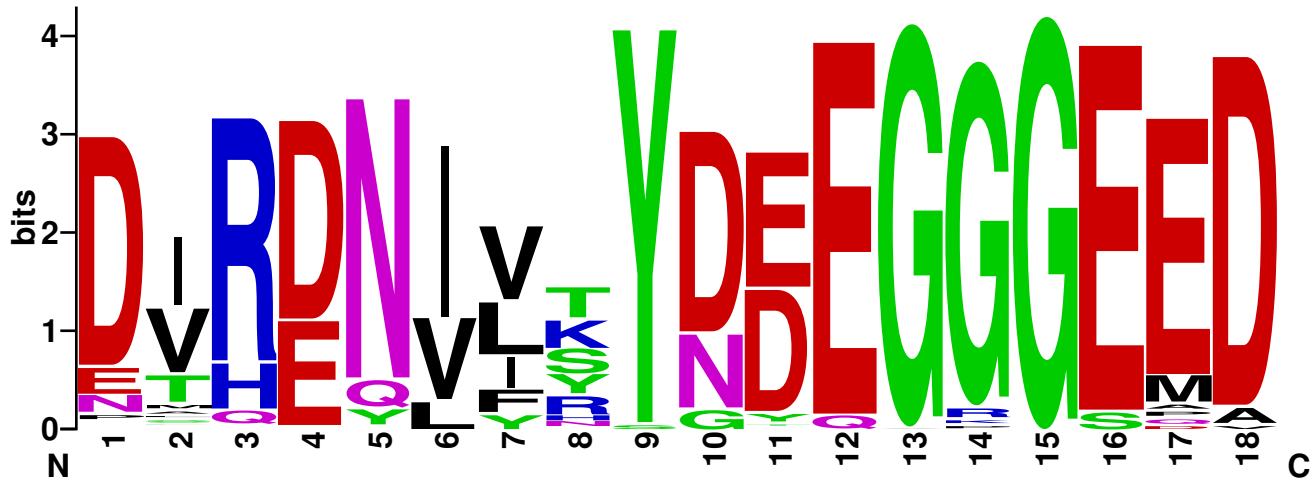

Supplement: Additional file 1 — Conserved Blocks that overlap with palindromes in proteins. [file 1471-2105-9-274-S1.zip › Logo/1I7WB.pdf]

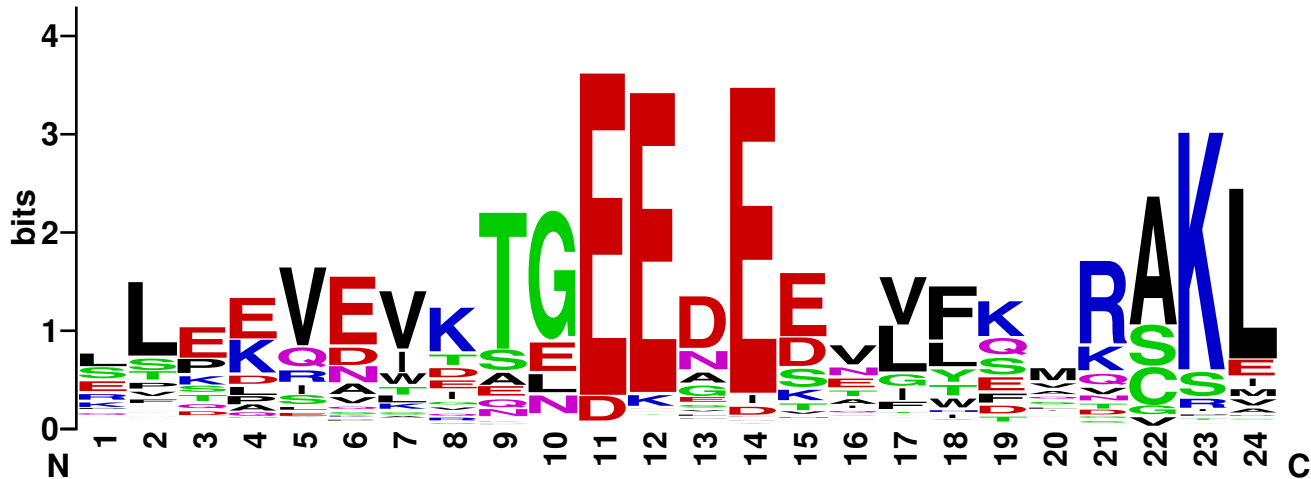

Supplement: Additional file 1 — Conserved Blocks that overlap with palindromes in proteins. [file 1471-2105-9-274-S1.zip › Logo/1K5DB.pdf]

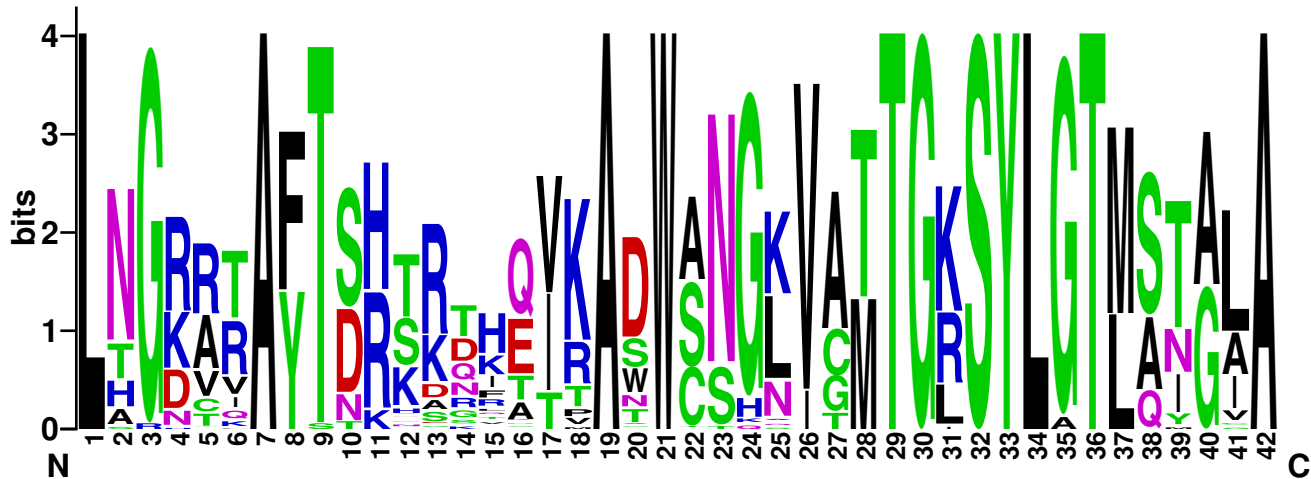

Supplement: Additional file 1 — Conserved Blocks that overlap with palindromes in proteins. [file 1471-2105-9-274-S1.zip › Logo/1LNSA.pdf]

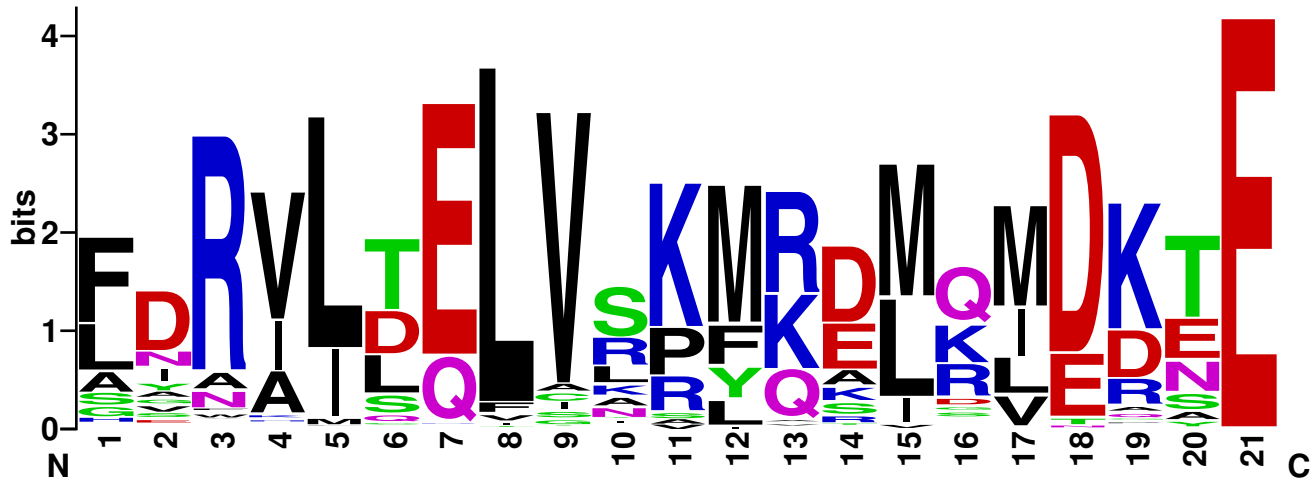

Supplement: Additional file 1 — Conserved Blocks that overlap with palindromes in proteins. [file 1471-2105-9-274-S1.zip › Logo/1LV2A.pdf]

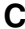

Supplement: Additional file 1 — Conserved Blocks that overlap with palindromes in proteins. [file 1471-2105-9-274-S1.zip › Logo/1MG4A.pdf]

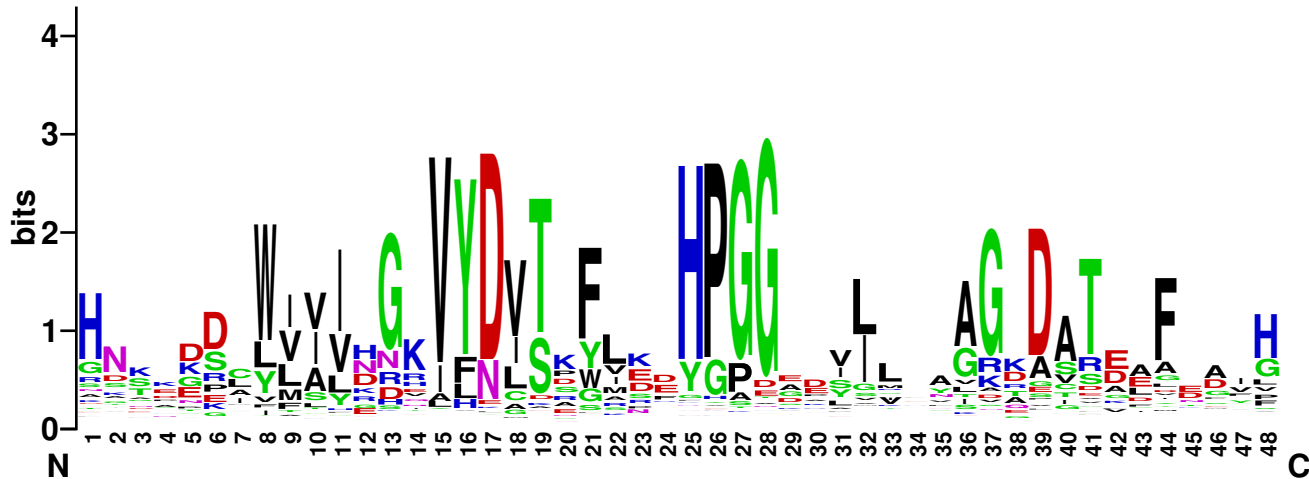

Supplement: Additional file 1 — Conserved Blocks that overlap with palindromes in proteins. [file 1471-2105-9-274-S1.zip › Logo/1MJ4A.pdf]

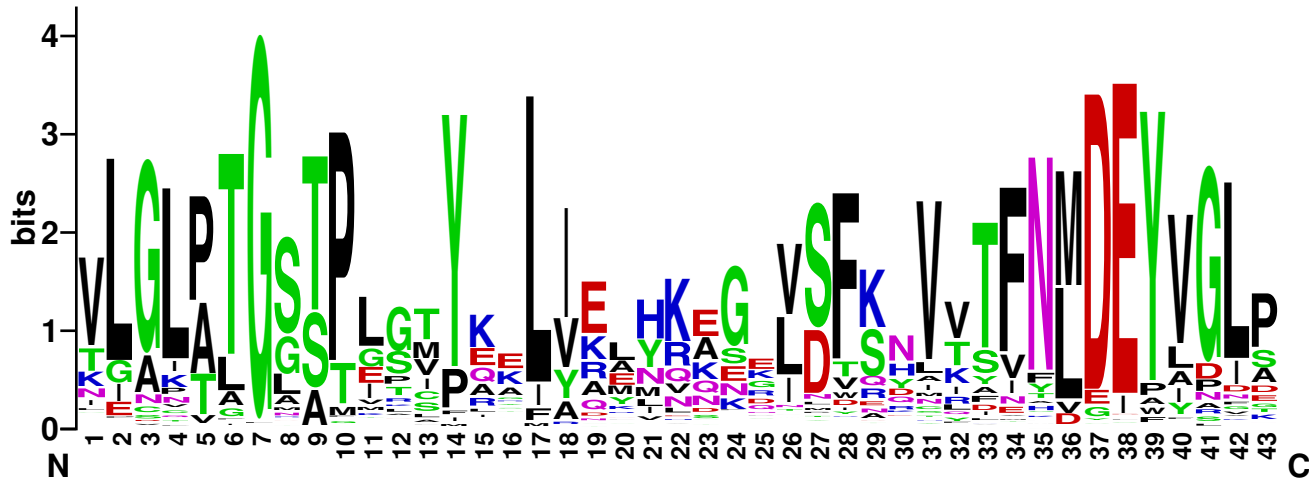

Supplement: Additional file 1 — Conserved Blocks that overlap with palindromes in proteins. [file 1471-2105-9-274-S1.zip › Logo/1NE7A.pdf]

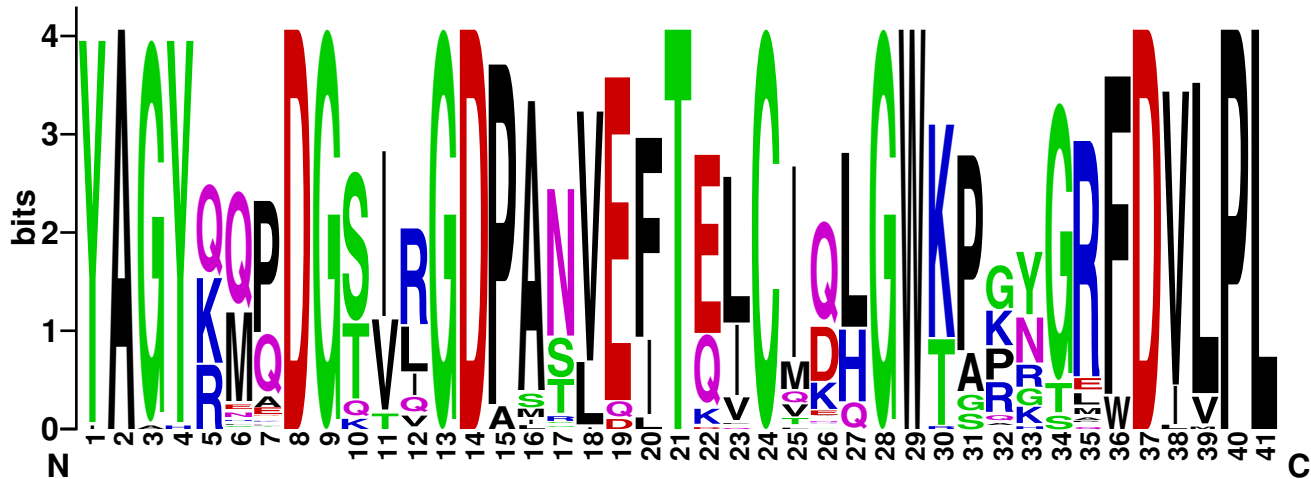

Supplement: Additional file 1 — Conserved Blocks that overlap with palindromes in proteins. [file 1471-2105-9-274-S1.zip › Logo/1OM4A.pdf]

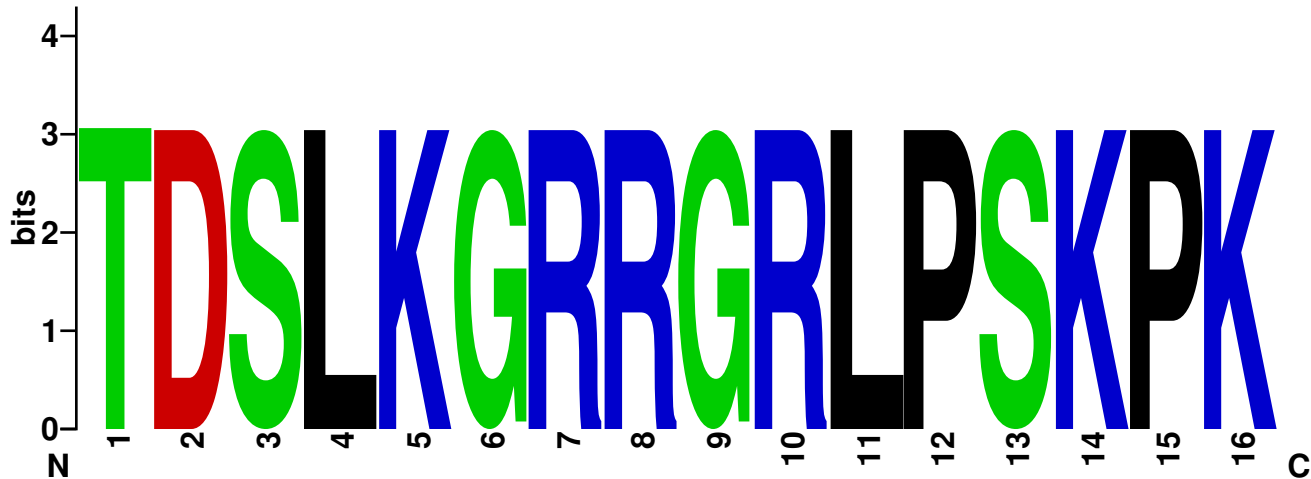

Supplement: Additional file 1 — Conserved Blocks that overlap with palindromes in proteins. [file 1471-2105-9-274-S1.zip › Logo/1OVLA.pdf]

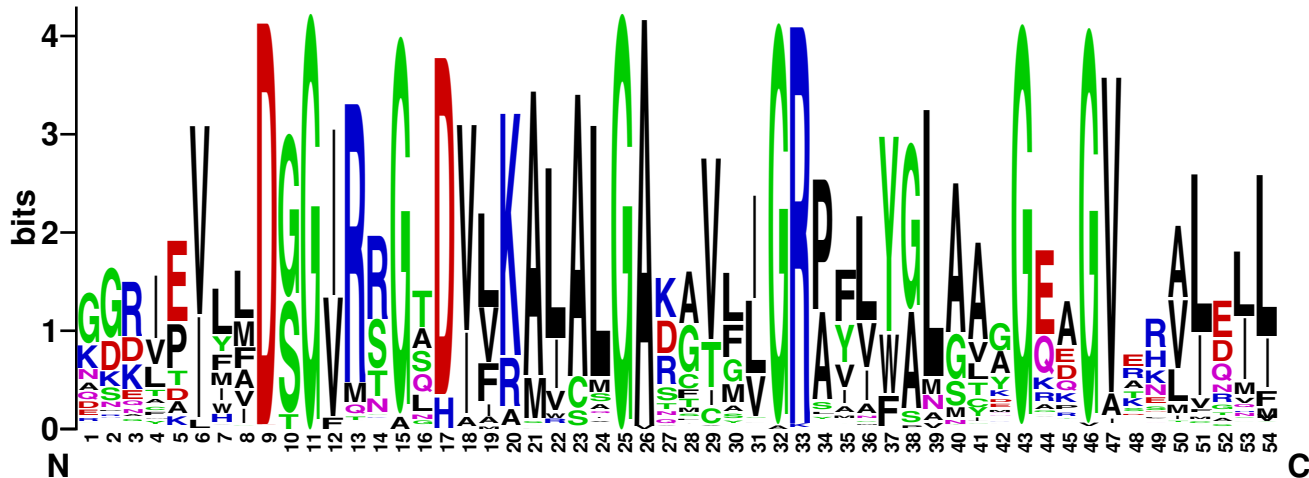

Supplement: Additional file 1 — Conserved Blocks that overlap with palindromes in proteins. [file 1471-2105-9-274-S1.zip › Logo/1P4CA, IDSGFRRGSDI.pdf]

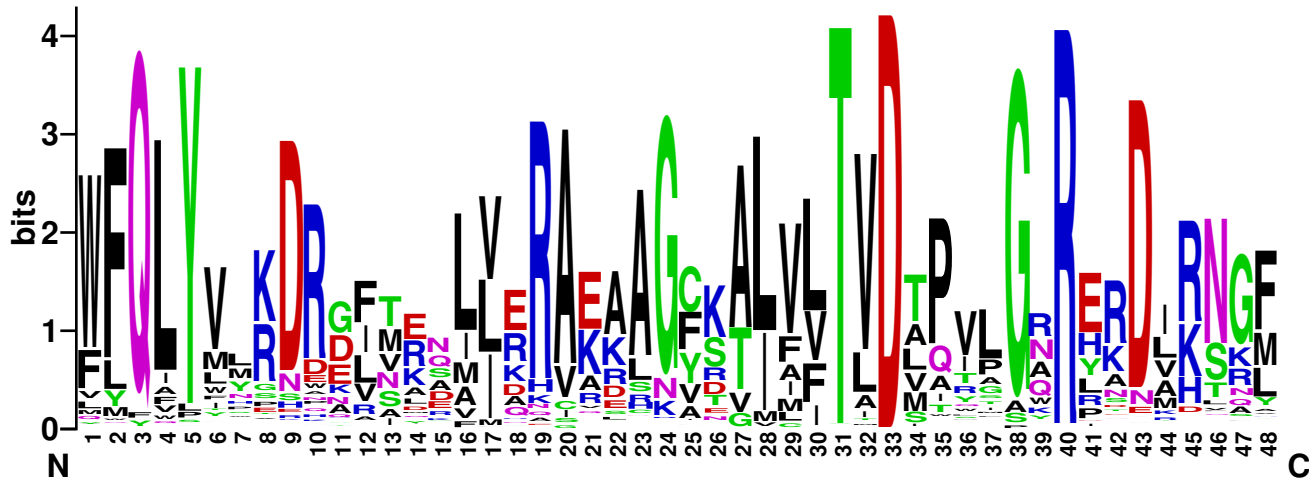

Supplement: Additional file 1 — Conserved Blocks that overlap with palindromes in proteins. [file 1471-2105-9-274-S1.zip › Logo/1P4CA, TTLVLTT.pdf]

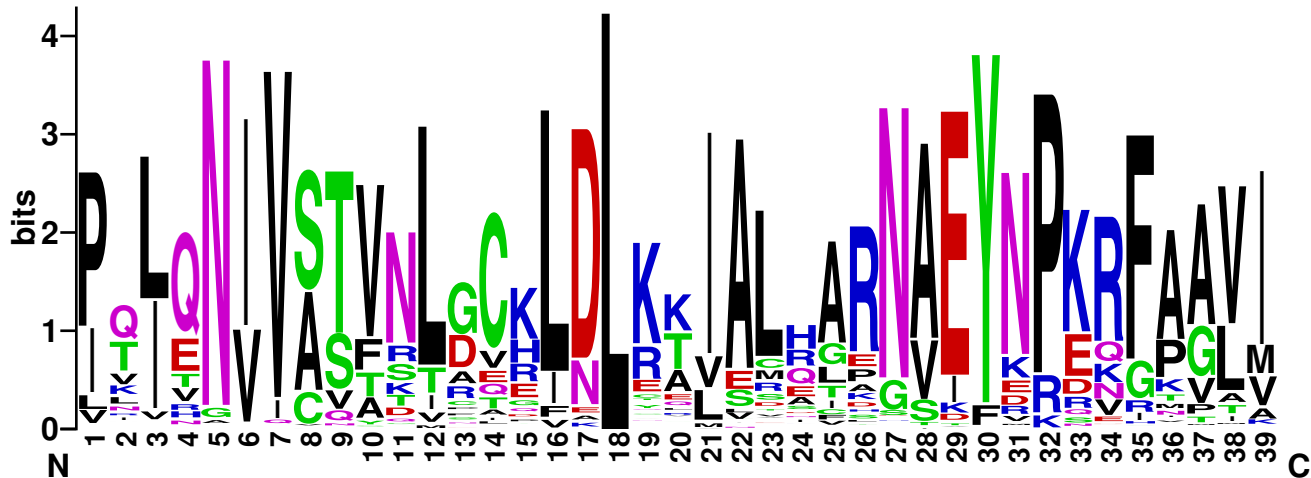

Supplement: Additional file 1 — Conserved Blocks that overlap with palindromes in proteins. [file 1471-2105-9-274-S1.zip › Logo/1PCZA.pdf]

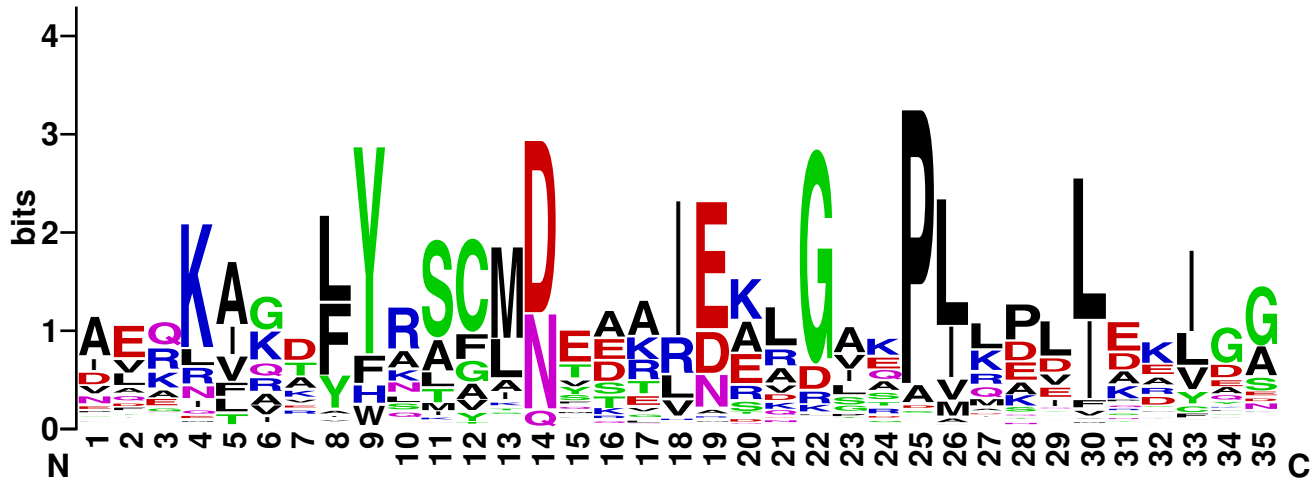

Supplement: Additional file 1 — Conserved Blocks that overlap with palindromes in proteins. [file 1471-2105-9-274-S1.zip › Logo/1R1HA.pdf]

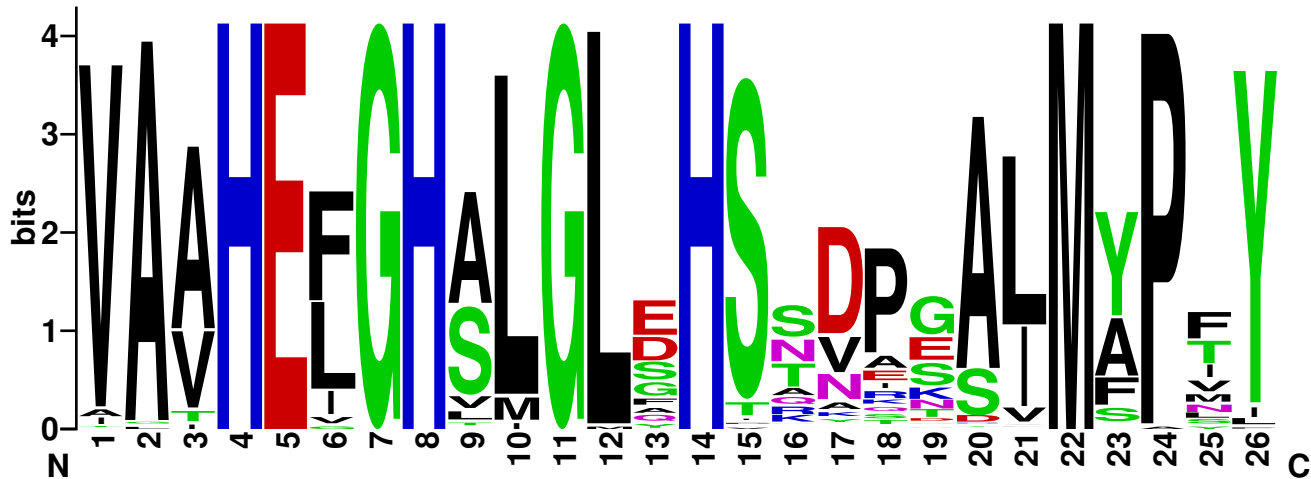

Supplement: Additional file 1 — Conserved Blocks that overlap with palindromes in proteins. [file 1471-2105-9-274-S1.zip › Logo/1RM8A.pdf]

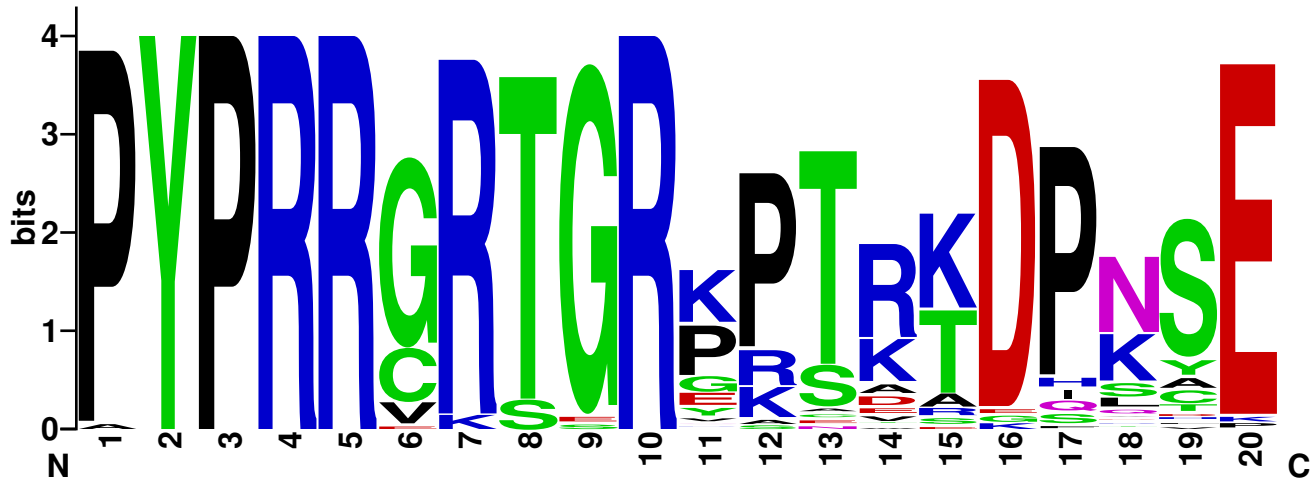

Supplement: Additional file 1 — Conserved Blocks that overlap with palindromes in proteins. [file 1471-2105-9-274-S1.zip › Logo/1RRHA.pdf]

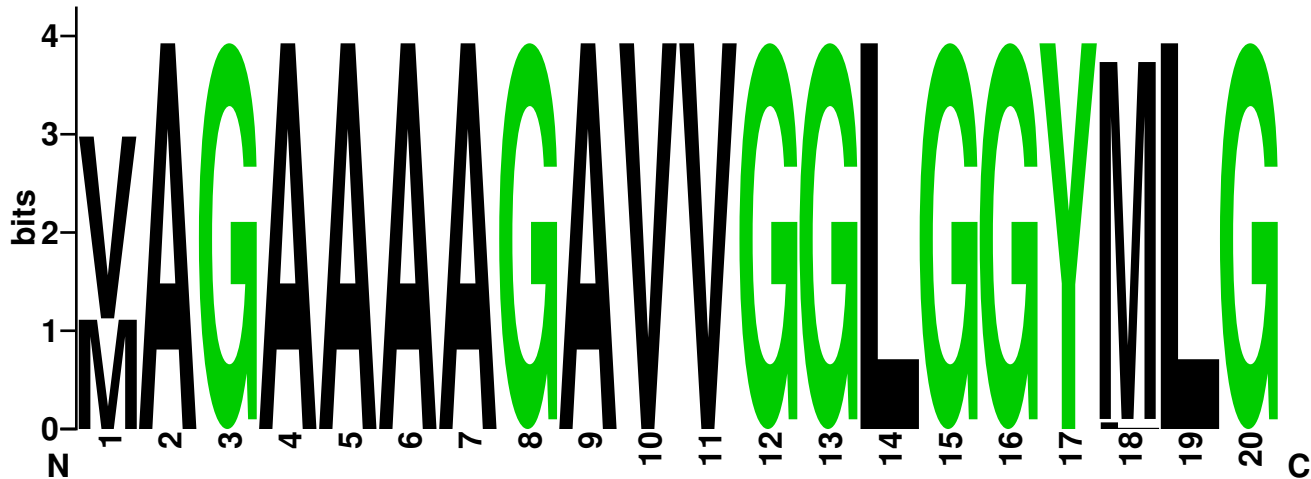

Supplement: Additional file 1 — Conserved Blocks that overlap with palindromes in proteins. [file 1471-2105-9-274-S1.zip › Logo/1TPXA.pdf]

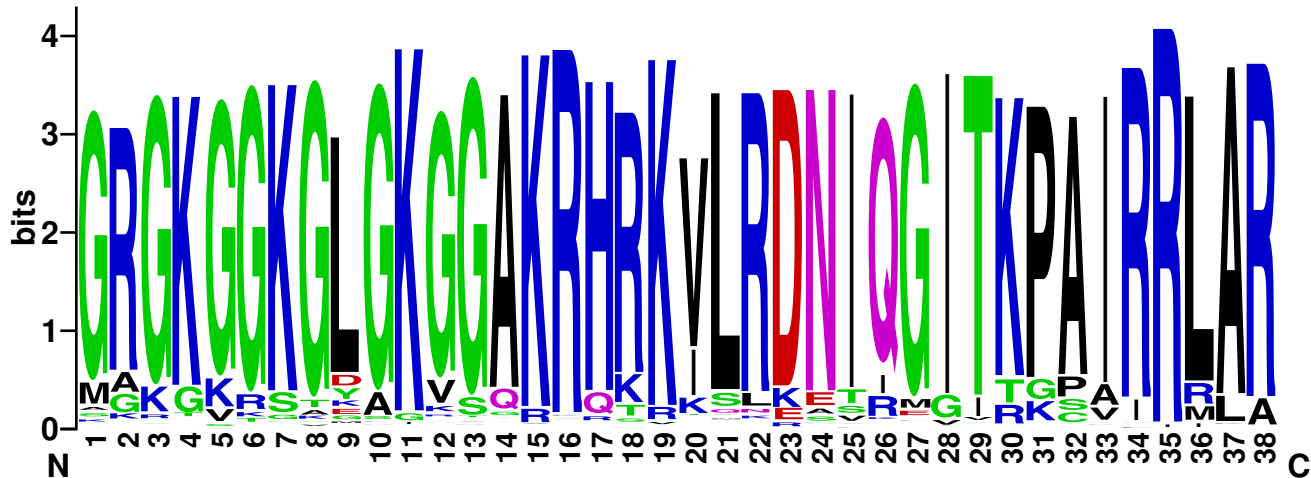

Supplement: Additional file 1 — Conserved Blocks that overlap with palindromes in proteins. [file 1471-2105-9-274-S1.zip › Logo/1TZYD, GGKGLGKGG.pdf]

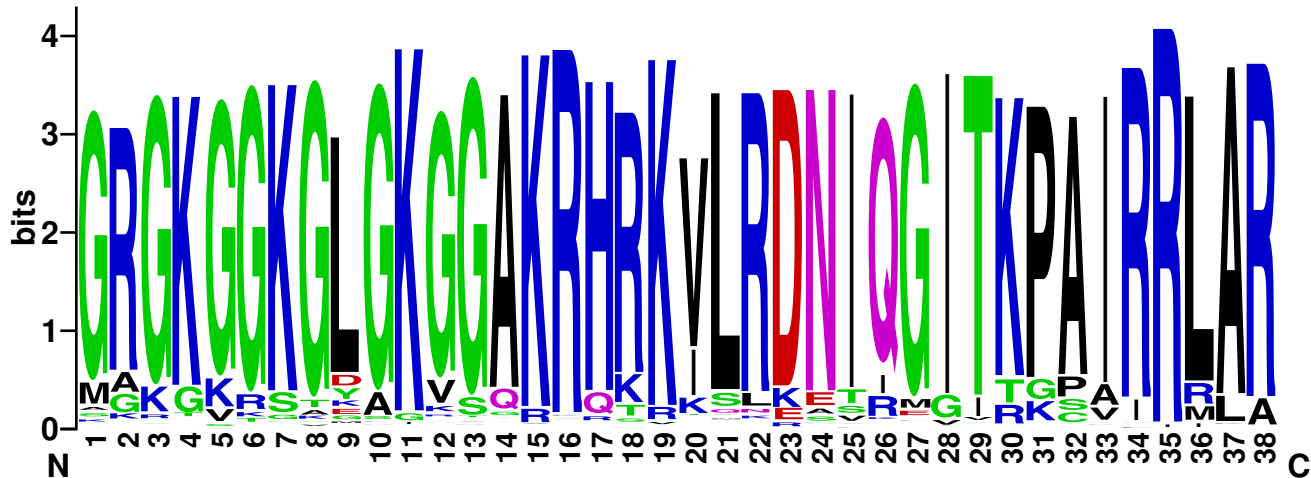

Supplement: Additional file 1 — Conserved Blocks that overlap with palindromes in proteins. [file 1471-2105-9-274-S1.zip › Logo/1TZYD, GRGKGGKG.pdf]

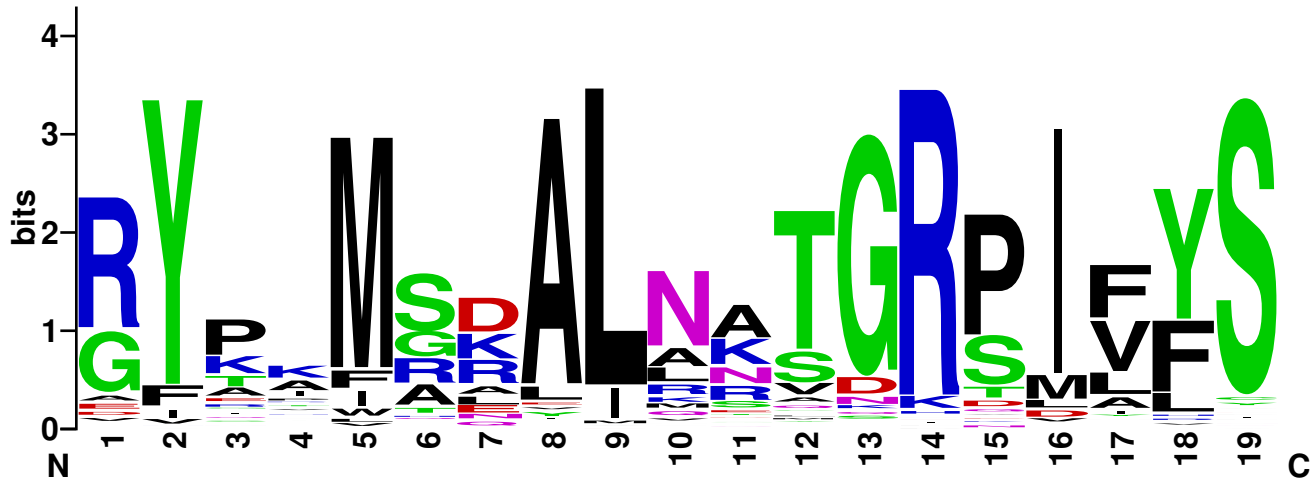

Supplement: Additional file 1 — Conserved Blocks that overlap with palindromes in proteins. [file 1471-2105-9-274-S1.zip › Logo/1UASA.pdf]

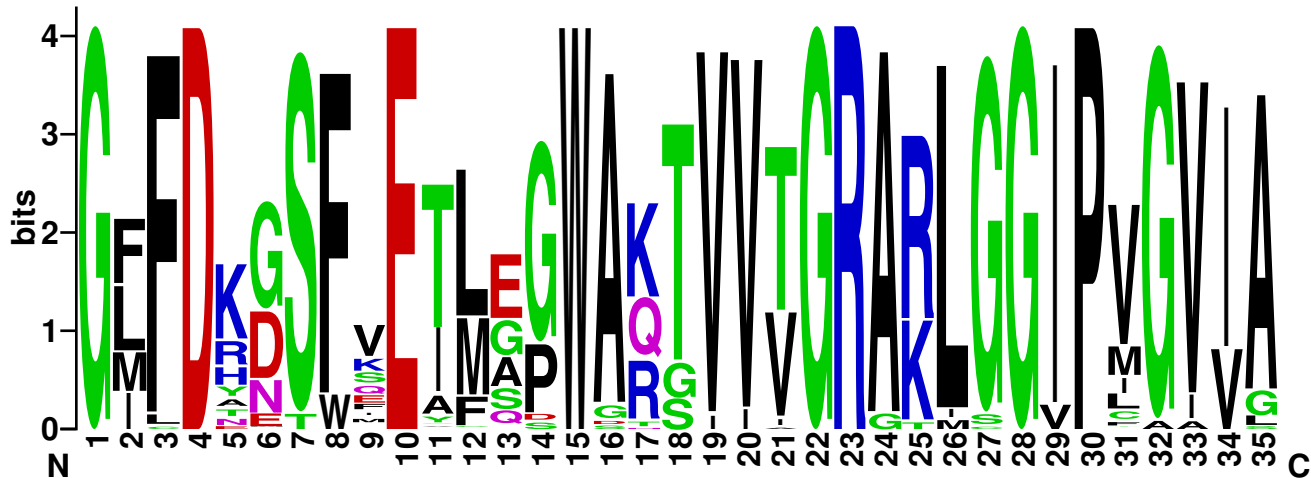

Supplement: Additional file 1 — Conserved Blocks that overlap with palindromes in proteins. [file 1471-2105-9-274-S1.zip › Logo/1UYRA.pdf]

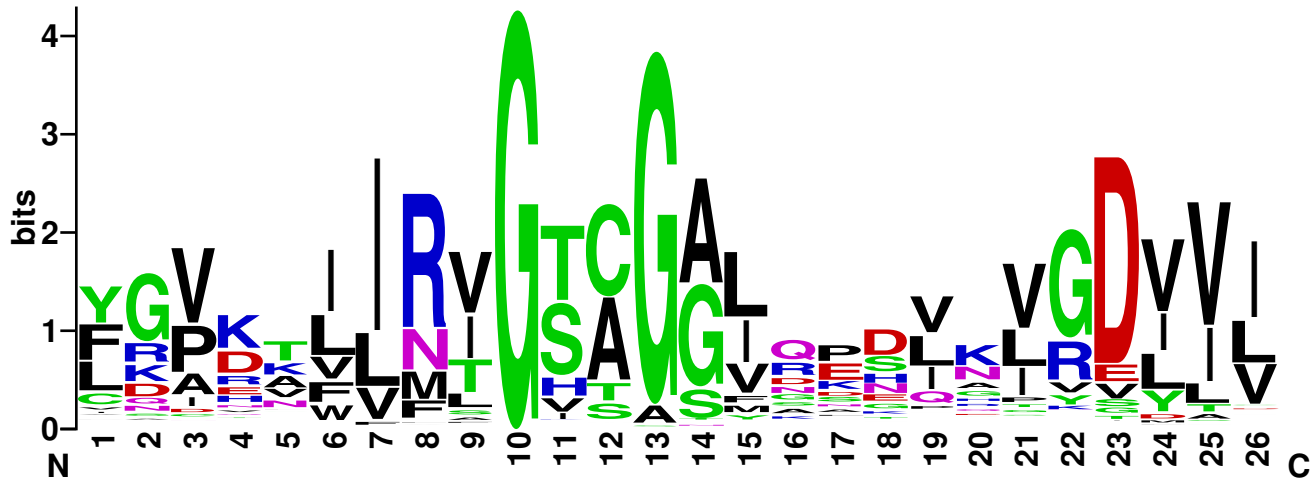

Supplement: Additional file 1 — Conserved Blocks that overlap with palindromes in proteins. [file 1471-2105-9-274-S1.zip › Logo/1VHWA.pdf]

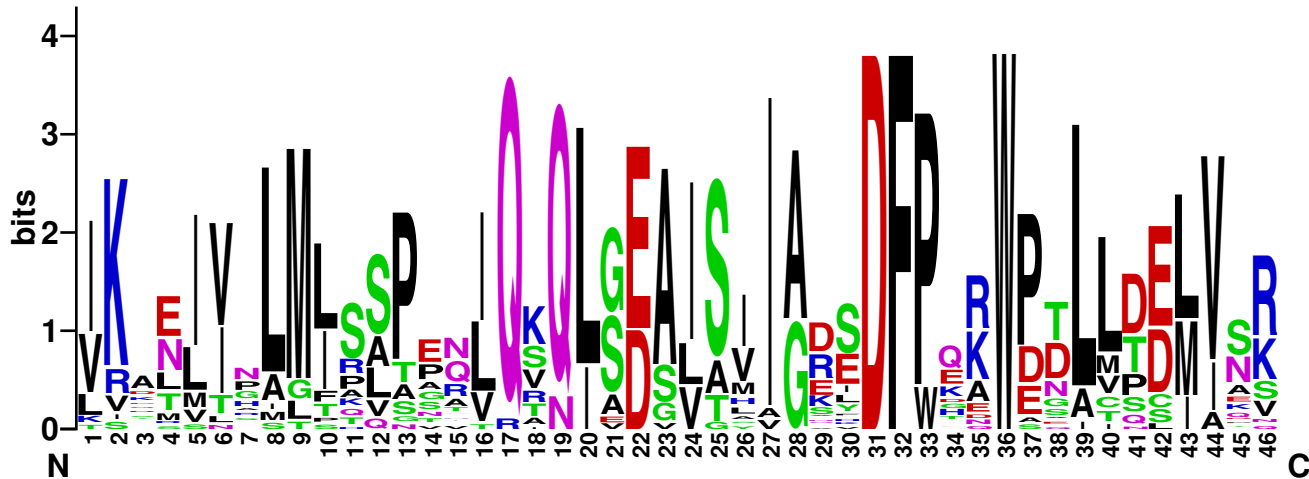

Supplement: Additional file 1 — Conserved Blocks that overlap with palindromes in proteins. [file 1471-2105-9-274-S1.zip › Logo/1WA5C.pdf]

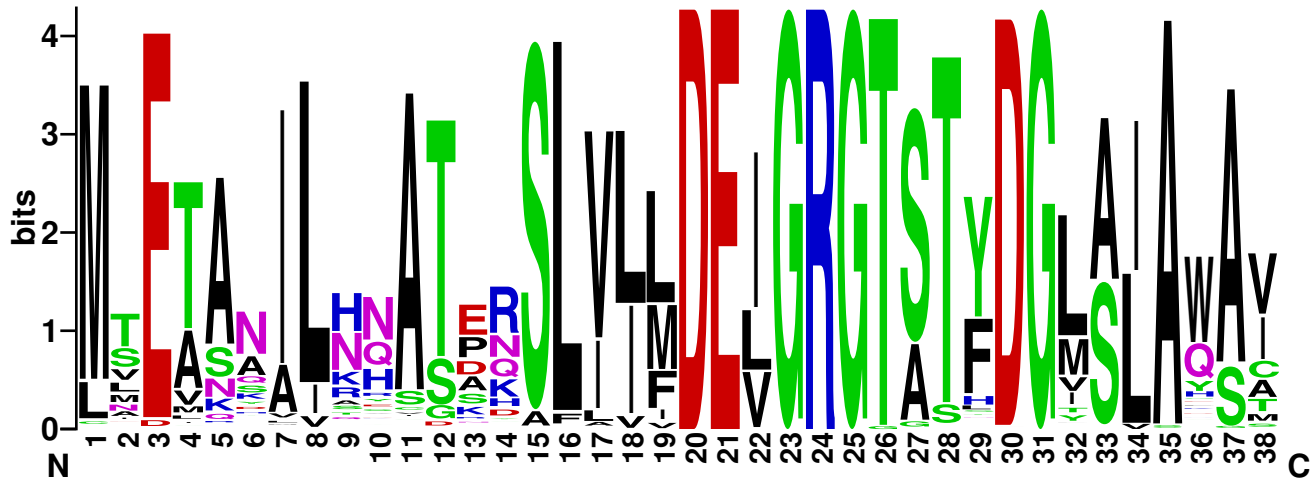

Supplement: Additional file 1 — Conserved Blocks that overlap with palindromes in proteins. [file 1471-2105-9-274-S1.zip › Logo/1WB9A.pdf]

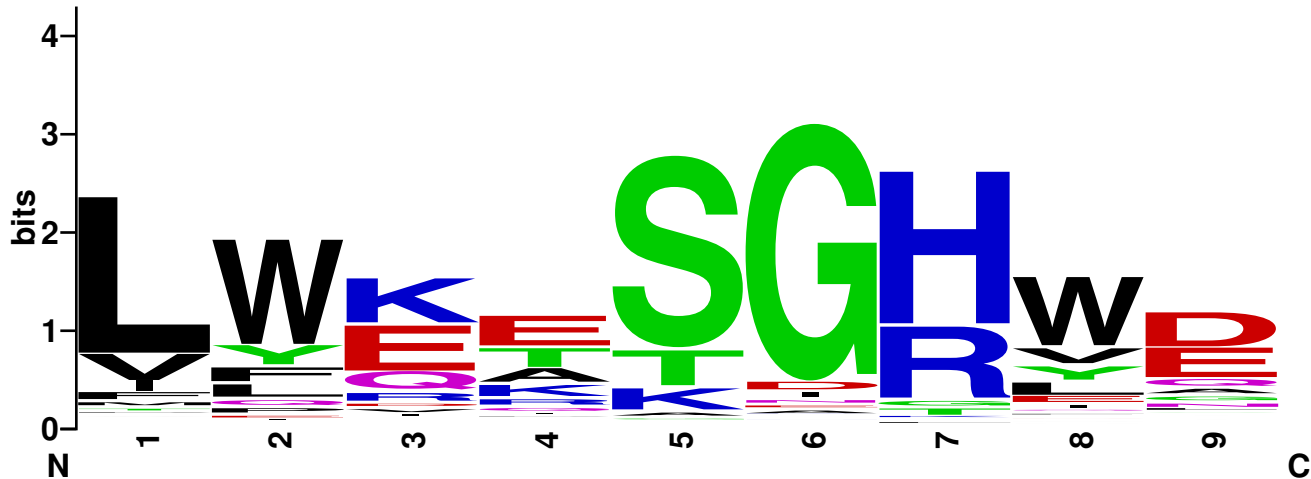

Supplement: Additional file 1 — Conserved Blocks that overlap with palindromes in proteins. [file 1471-2105-9-274-S1.zip › Logo/1WU7A.pdf]

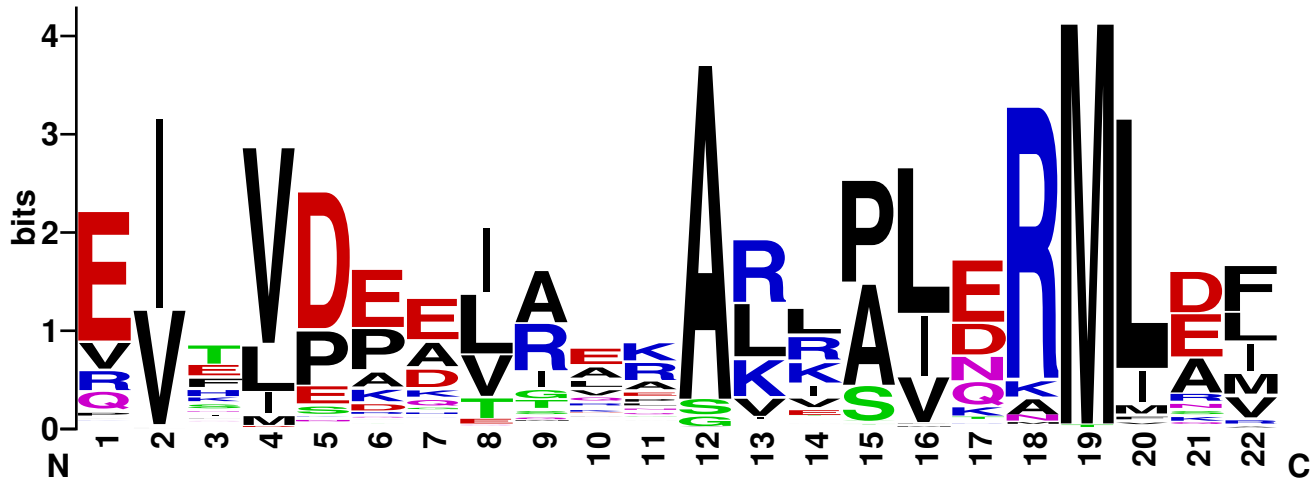

Supplement: Additional file 1 — Conserved Blocks that overlap with palindromes in proteins. [file 1471-2105-9-274-S1.zip › Logo/1WZUA.pdf]

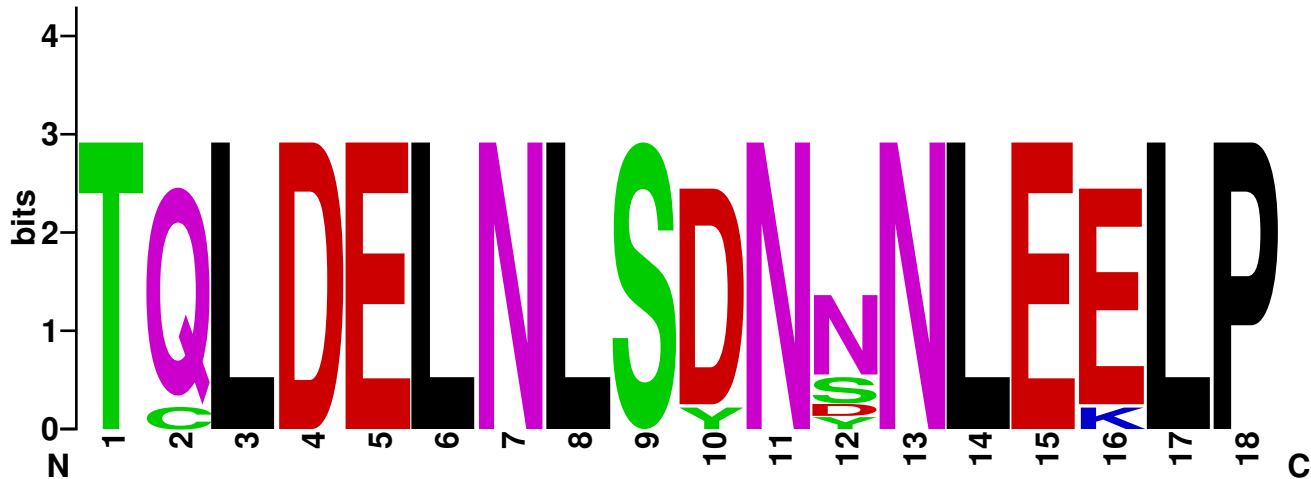

Supplement: Additional file 1 — Conserved Blocks that overlap with palindromes in proteins. [file 1471-2105-9-274-S1.zip › Logo/1XWDC.pdf]

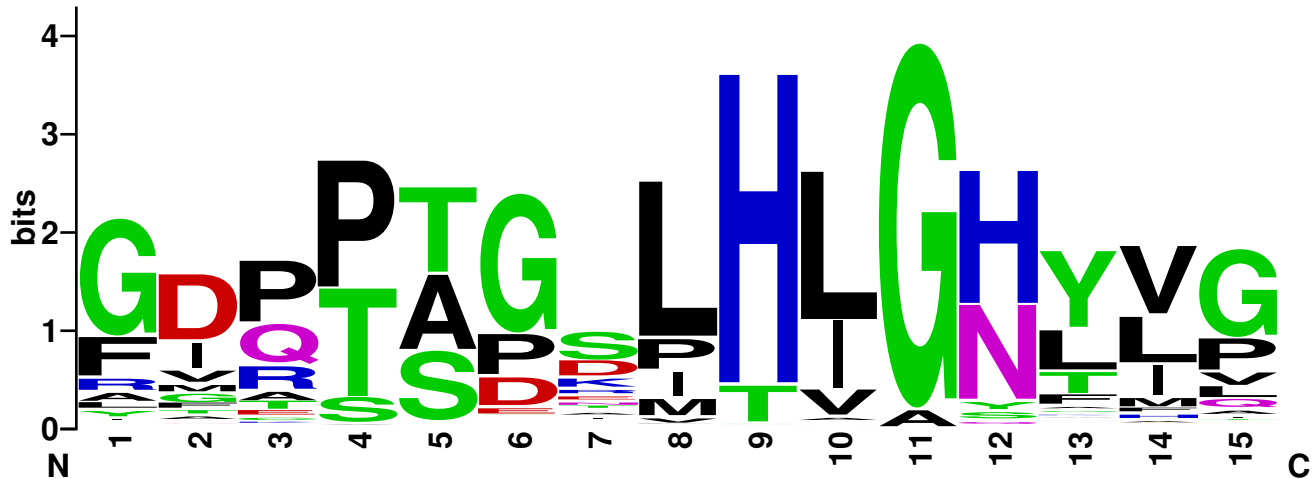

Supplement: Additional file 1 — Conserved Blocks that overlap with palindromes in proteins. [file 1471-2105-9-274-S1.zip › Logo/1YI8A.pdf]

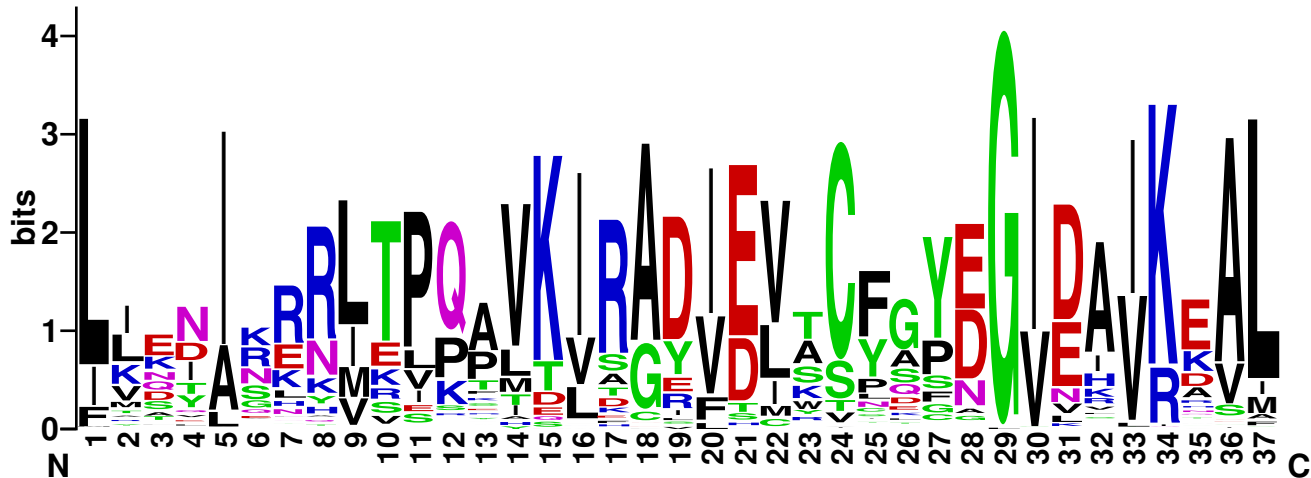

Supplement: Additional file 1 — Conserved Blocks that overlap with palindromes in proteins. [file 1471-2105-9-274-S1.zip › Logo/1YZ7A.pdf]

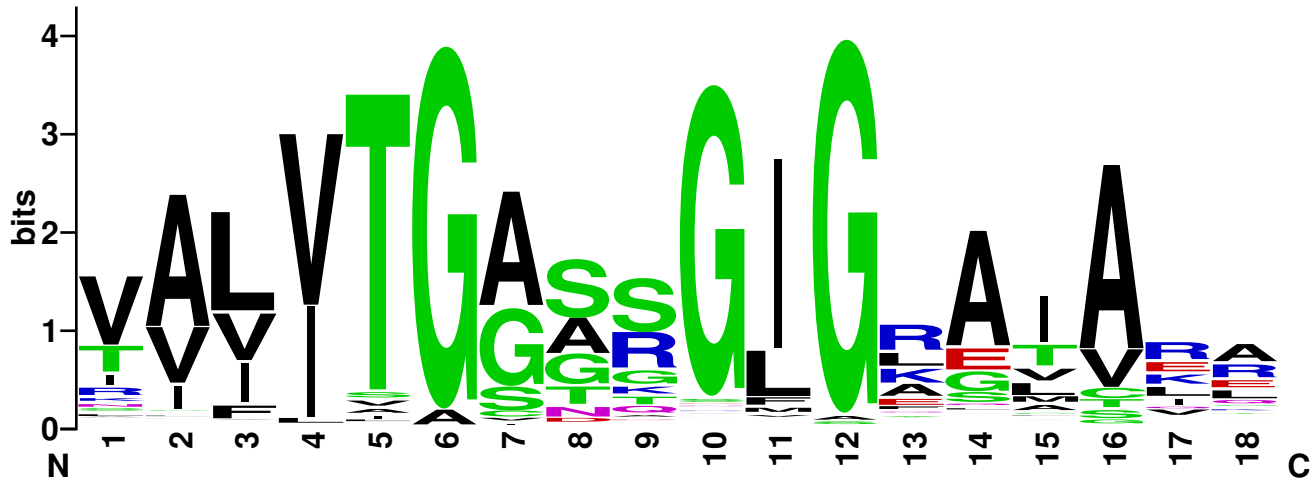

Supplement: Additional file 1 — Conserved Blocks that overlap with palindromes in proteins. [file 1471-2105-9-274-S1.zip › Logo/2AG5A.pdf]

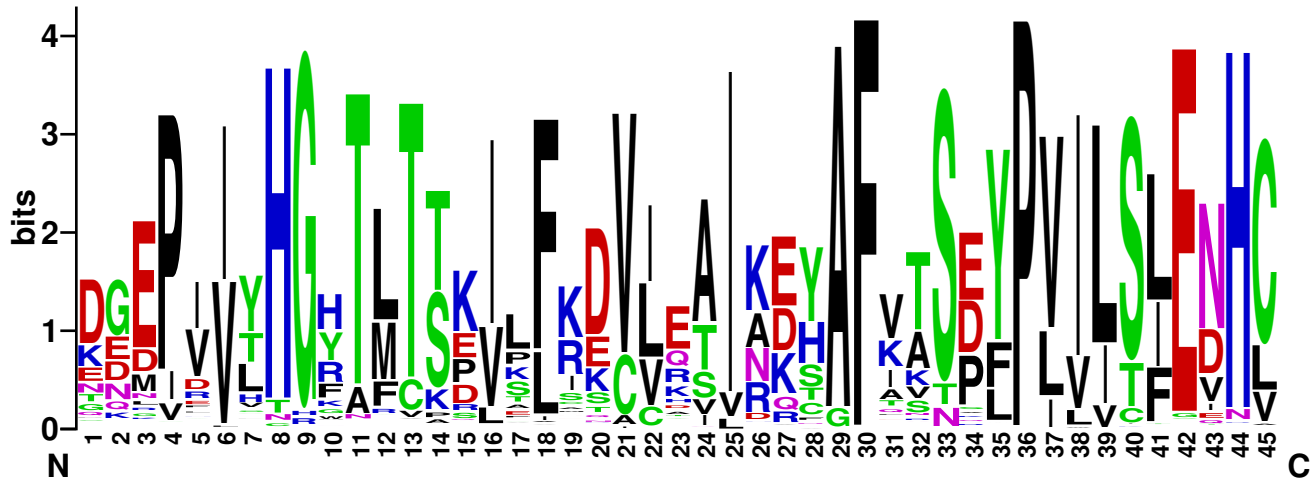

Supplement: Additional file 1 — Conserved Blocks that overlap with palindromes in proteins. [file 1471-2105-9-274-S1.zip › Logo/2FJUB.pdf]

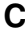

Supplement: Additional file 1 — Conserved Blocks that overlap with palindromes in proteins. [file 1471-2105-9-274-S1.zip › Logo/2FP4B.pdf]

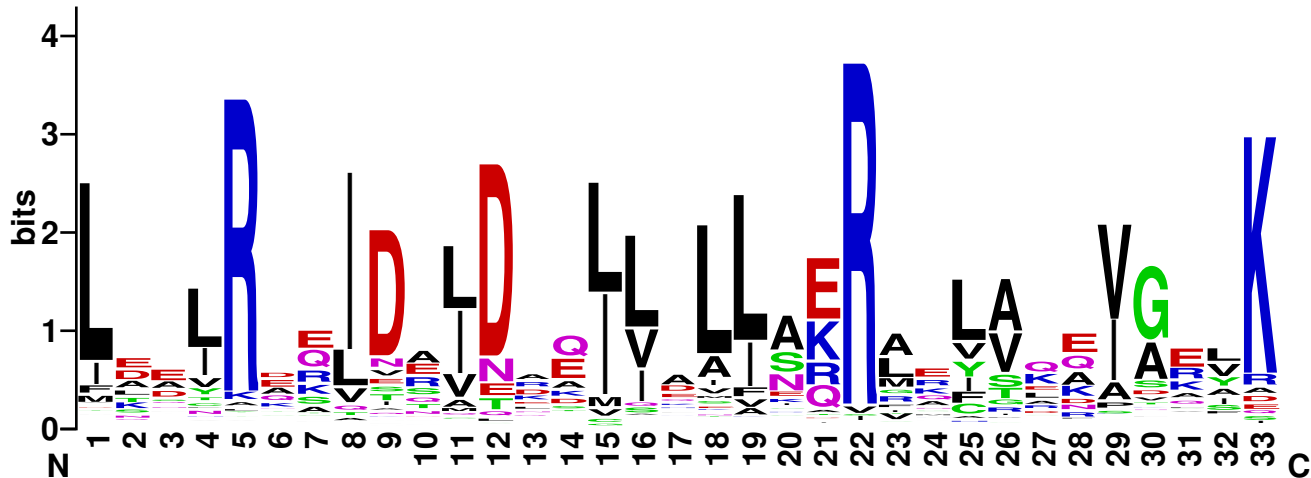

Supplement: Additional file 1 — Conserved Blocks that overlap with palindromes in proteins. [file 1471-2105-9-274-S1.zip › Logo/2H9DA.pdf]

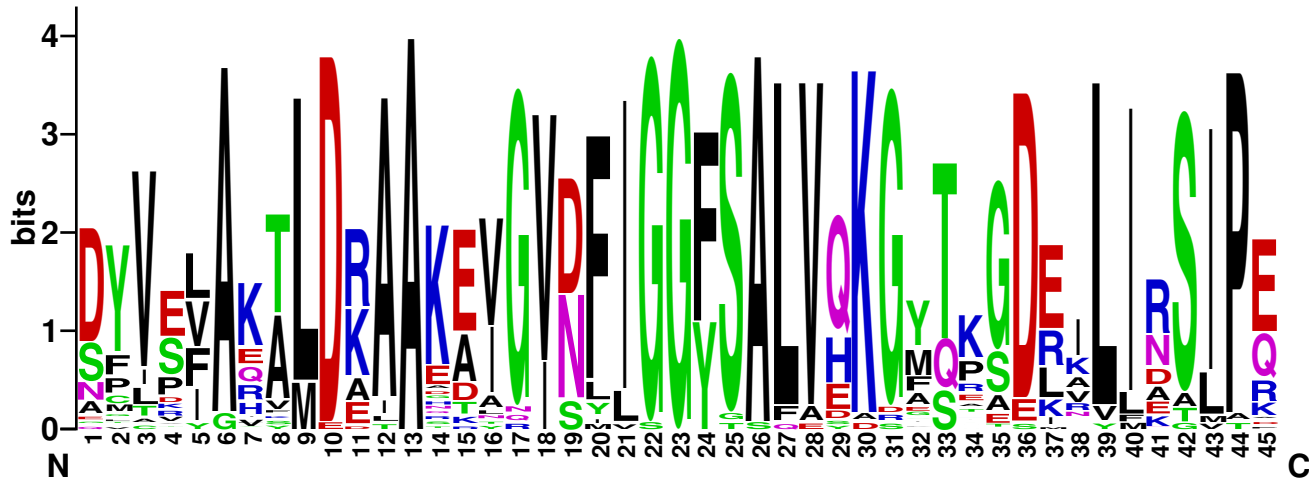

Supplement: Additional file 1 — Conserved Blocks that overlap with palindromes in proteins. [file 1471-2105-9-274-S1.zip › Logo/2HA9A, DKAAKE.pdf]

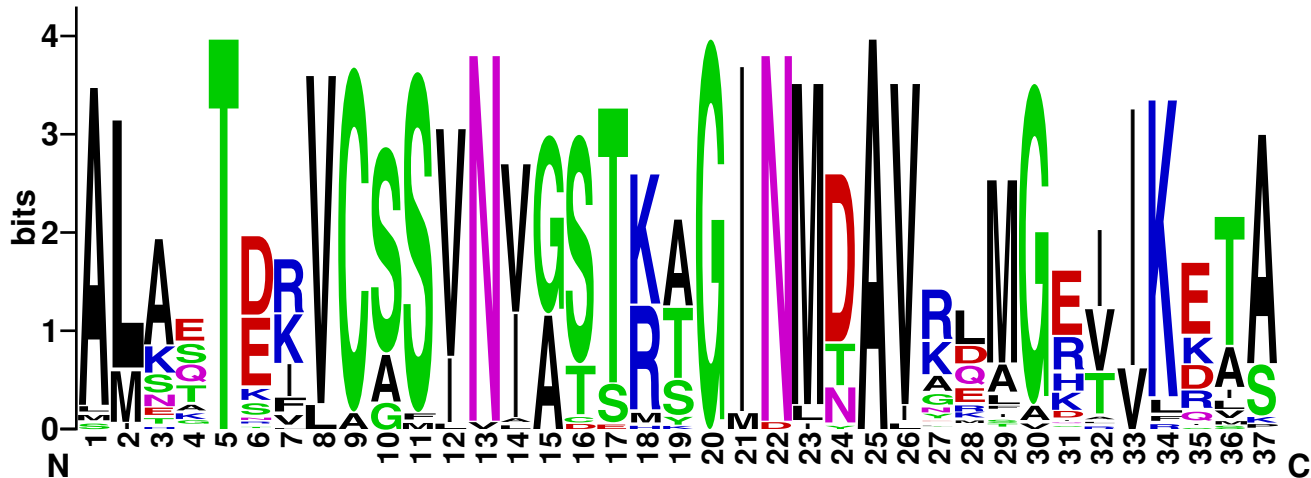

Supplement: Additional file 1 — Conserved Blocks that overlap with palindromes in proteins. [file 1471-2105-9-274-S1.zip › Logo/2HA9A, NIGSTKSGIN.pdf]

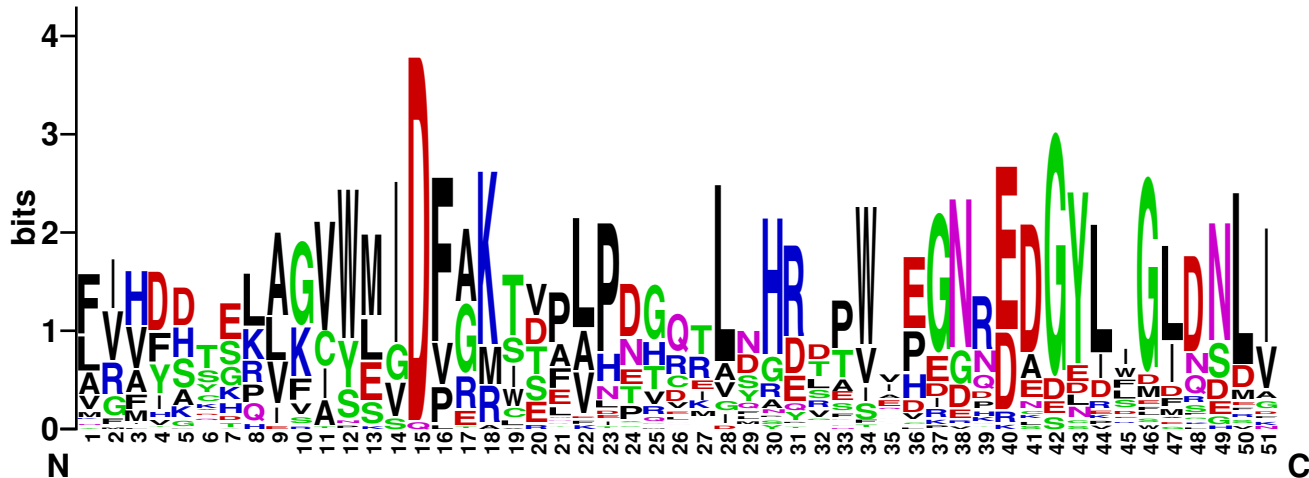

Supplement: Additional file 1 — Conserved Blocks that overlap with palindromes in proteins. [file 1471-2105-9-274-S1.zip › Logo/2IEWA.pdf]
