# Supplementary material for: A tale of two symmetrical tails: Structural and functional characteristics of palindromes in proteins
Source: BMC Bioinformatics. 2008 Jun 11;9:274. doi: 10.1186/1471-2105-9-274 (PMC2474621; doi:10.1186/1471-2105-9-274)
Supplement: Additional file 1 — Conserved Blocks that overlap with palindromes in proteins. [file 1KV0A.pdf › Logo/1KV0A.pdf]

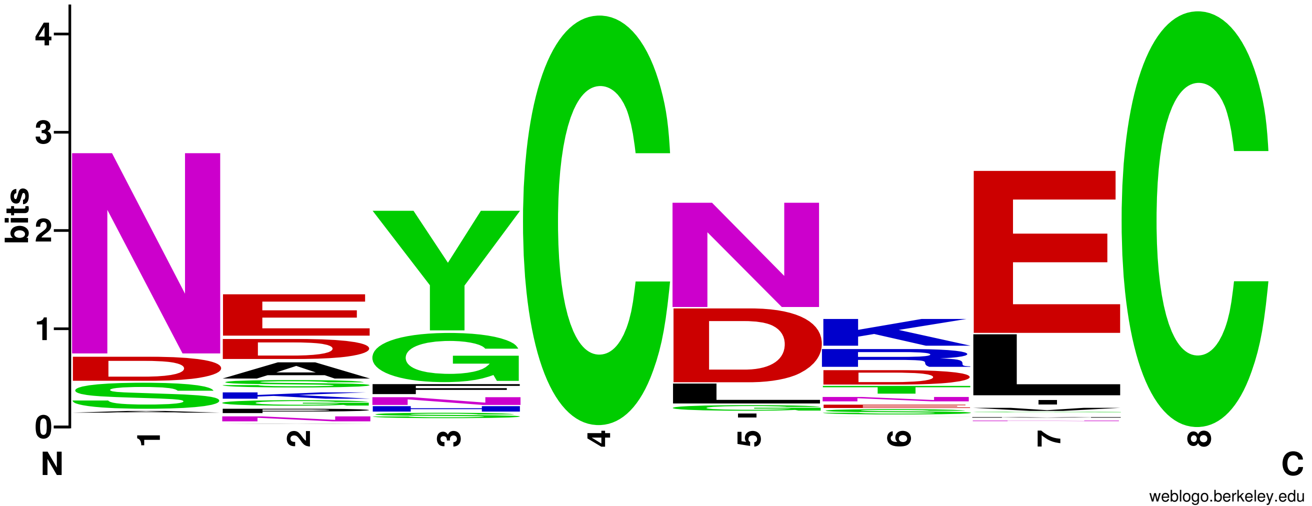

bits
0
1
2
3
4
N
1
2
3
4
5
6
7
8
C
weblogo.berkeley.edu
